# Supplementary figures and images for: Proportionality: A Valid Alternative to Correlation for Relative Data
Source: PLoS Comput Biol. 2015 Mar 16;11(3):e1004075. doi: 10.1371/journal.pcbi.1004075 (PMC4361748; doi:10.1371/journal.pcbi.1004075)

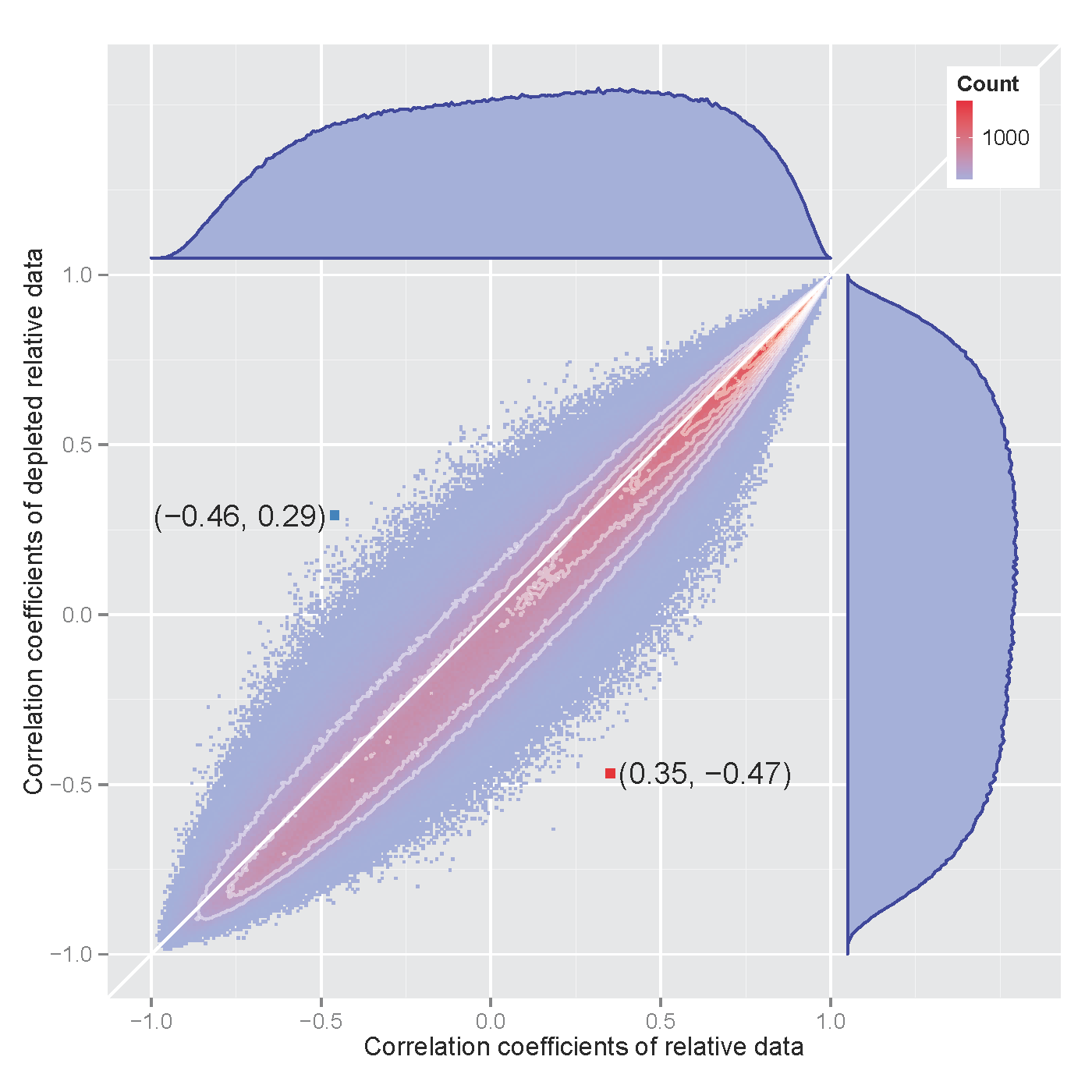

Supplement: S3 Fig — White contour lines are shown at intervals of 100 counts. While the distribution of the correlation coefficient pairs lies more on the diagonal than in the preceding figure, it is clear that correlation of relative abundances is sensitive to what is in (or out of) the total, i.e., correlation is not subcompositionally coherent. (TIFF) [file pcbi.1004075.s003.tiff]

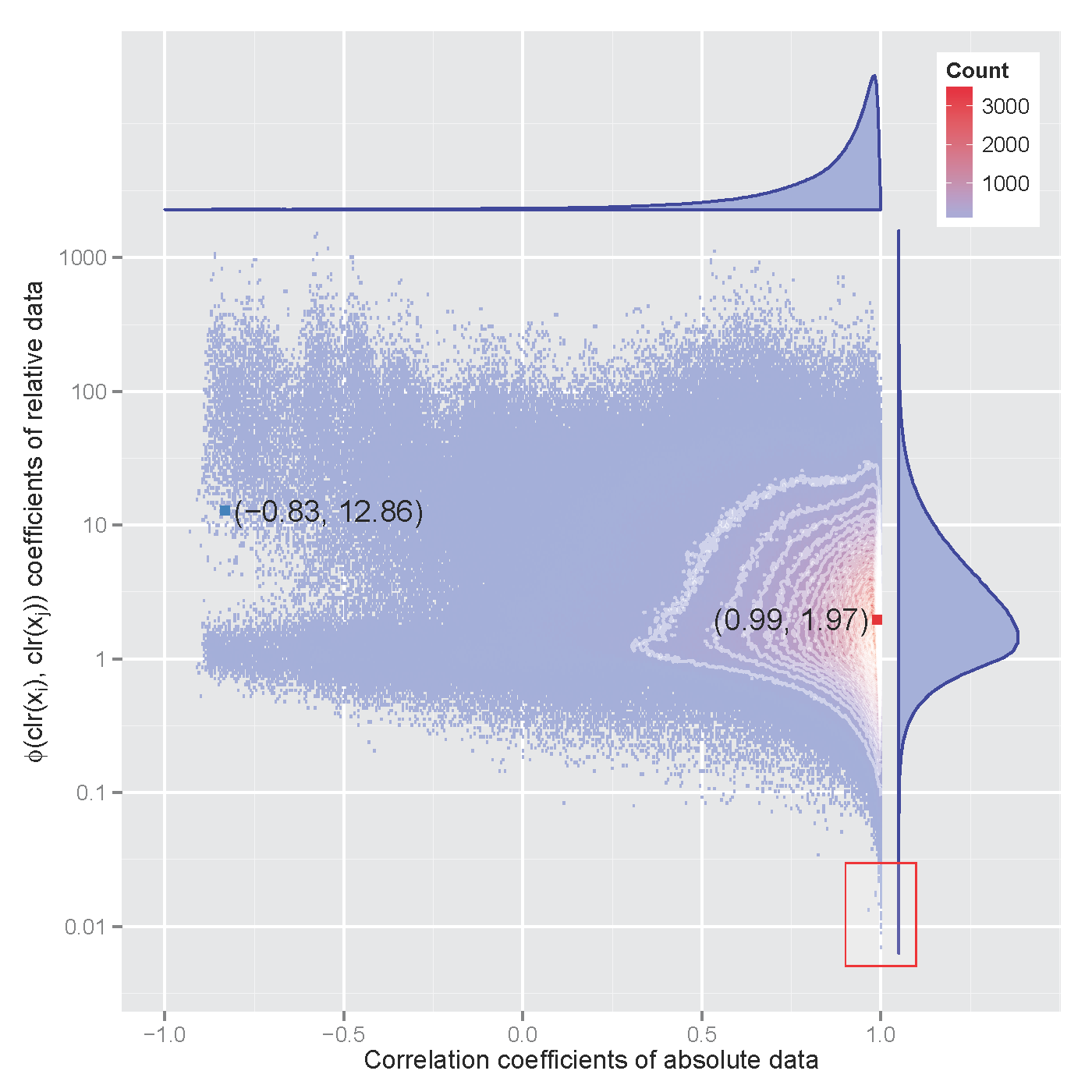

Supplement: S4 Fig — The red and blue points correspond to the red and blue pairs of mRNA in Fig. 2. White contour lines are shown at intervals of 100 counts and the top marginal histogram is the same as in S2(b) Fig. The few mRNA pairs that are strongly proportional (within the red rectangle) are also strongly positively correlated. However, the converse is not true: strong positive correlation between mRNAs does not imply that they are strongly proportional. (TIFF) [file pcbi.1004075.s004.tiff]
