# Supplementary material for: Proportionality: A Valid Alternative to Correlation for Relative Data
Source: PLoS Comput Biol. 2015 Mar 16;11(3):e1004075. doi: 10.1371/journal.pcbi.1004075 (PMC4361748; doi:10.1371/journal.pcbi.1004075)
Supplement: S1 Supporting Information — This PDF file is the output obtained by executing SupplementaryInfo.Rnw from S2 Supporting Information. In addition to all the figures and results in the manuscript it provides additional detail and information for those interested in understanding more about compositional data analysis and the analyses we have conducted. (PDF) [file pcbi.1004075.s011.pdf]

Proportionality: a valid alternative to correlation for relative data: Supplementary Information

David Lovell[1,*], Vera Pawlowsky-Glahn[2], Juan José Egozcue[3], Samuel Marguerat[4], Jürg Bähler[5]

October 1, 2014

1. CSIRO Computational Informatics, Canberra, Australia.
2. Dept. d'Informàtica, Matemàtica Aplicada i Estadística. U. de Girona, España
3. Dept. Applied Mathematics III, U. Politécnica de Catalunya, Barcelona, Spain.
4. MRC Clinical Sciences Centre, Imperial College London, United Kingdom
5. Research Department of Genetics, Evolution and Environment, University College London, United Kingdom.
* Corresponding author David Lovell (David.Lovell@csiro.au)

S1 Outline of this Supplementary Information

This document aims to

1. ensure the research reported in *Proportionality: a valid alternative to correlation for relative data* is reproducible. This document was produced in RStudio using `Sweave` [1] and the `knitr` package [2] from the file `SupplementaryInfo.Rnw` available in the Supplementary Information zip file.

2. provide additional detail, figures and information for those interested in understanding more about compositional data analysis and the analyses we have conducted.

This Supplementary Information is meant to be read in conjunction with the main paper and is broken into the following sections:

Why does compositional data need special treatment? gives examples to illustrate some of the problems that arise when analyses and interpretations ignore the relative nature of data.

Preparing the data for analysis explains key steps in preparing the yeast gene expression data of Marguerat et al. [3] for further analysis and visualisation.

Problems with analyses that ignore the relative nature of data demonstrates how correlation is an inappropriate and misleading measure of association for data that carry only relative information. It also shows how the concept of "differential expression" can be challenging to interpret when applied to relative abundances.

Measuring association as "goodness of fit to proportionality" explores $\phi()$, a well-founded alternative to correlation for data that carry only relative information. After showing how $\phi()$ depends on the slope and correlation of pairs of relative values, we show how it can be calculated efficiently in `R` then used as a basis for analyses and visualisations that are familiar in molecular bioscience.

On the mathematics of different representations discusses the mathematical reasoning behind the logarithmic and centred logratio representations of data.

Pombase information on mRNAs behaving proportionally tabulates descriptions of the clusters of yeast genes that showed proportional levels of expression in our analysis of [3].

S1.1 Executing this Supplementary Information

This document (`SupplementaryInfo.pdf`) was created using `R` version 3.0.2 (2013-09-25) via RStudio. (Note that the `compositions` package [4] is not yet available under `R` version 3.0.3). To re-execute the analysis described in this document,

1. Install RStudio and ensure it is running `R` version 3.0.2
2. Ensure the following packages are installed: [5, 6, 7, 4, 8, 9, 10, 11, 2, 12, 13, 14, 15, 16, 17, 18, 19, 20, 21, 22]
3. In RStudio under `Ensure Project` → `Project Options` → `Sweave` set the option to "Weave Rnw files using: knitr"
4. Open `SupplementaryInfo.Rnw` in RStudio
5. Click "Compile PDF"

This complation will create the directory `./figures` which will contain all the figures used in this document, and the main manuscript.

S2 Why does compositional data need special treatment?

Here are three examples to illustrate some of the problems that arise when analyses and interpretations ignore the relative nature of data.

S2.1 Correlation is not subcompositionally coherent

For the reader's benefit, we reproduce Section 1.7 of Aitchison's *A Concise Guide to Compositional Data Analysis* [23] which provides a classic illustration of why correlation is an inappropriate measure of association for compositional data:

> Consider two scientists A and B interested in soil samples, which have been divided into aliquots For each aliquot A records a 4-part composition (animal, vegetable, mineral, water); B first dries each aliquot without recording the water content and arrives at a 3-part composition (animal, vegetable, mineral). Let us further assume for simplicity the ideal situation where the aliquots in each pair are identical and where the two scientists are accurate in their determinations. Then clearly B's 3-part composition $[s_1, s_2, s_3]$ for an aliquot will be a subcompositionof A's 4-part composition $[x_1, x_2, x_3, x_4]$ for the corresponding aliquot related as in the definition of subcomposition in Section 1.5 above with C = 3, D = 4. It is then obvious that any compositional statements that A and B make about the common parts, animal, vegetable and mineral, must agree. This is the nature of subcompositional coherence.
>
> The ignoring of this principle of subcompositional coherence has been a source of great confusion in compositional data analysis. The literature, even currently, is full of attempts to explain the dependence of components of compositions in terms of product moment correlation of raw components. Consider the simple data set:

```
Full.compositions

##   animal vegetable mineral water
## 1    0.1       0.2     0.1   0.6
## 2    0.2       0.1     0.1   0.6
## 3    0.3       0.3     0.2   0.2

Subcompositions

##   animal vegetable mineral
## 1  0.250     0.500    0.25
## 2  0.500     0.250    0.25
## 3  0.375     0.375    0.25

cor(Full.compositions)

##           animal vegetable mineral  water
## animal     1.000     0.500   0.866 -0.866
## vegetable  0.500     1.000   0.866 -0.866
## mineral    0.866     0.866   1.000 -1.000
## water     -0.866    -0.866  -1.000  1.000

cor(Subcompositions)

##           animal vegetable mineral
## animal         1        -1      NA
## vegetable     -1         1      NA
## mineral       NA        NA       1
```

Scientist A would report the correlation between animal and vegetable as $\rho(x_1, x_2) = 0.5$ whereas B would report $\rho(s_1, s_2) = -1$. There is thus incoherence of the product-moment correlation between raw components as a measure of dependence. Note, however, that the ratio of two components remains unchanged when we move from full composition to subcomposition: $s_i/s_j = x_i/x_j$, so that as long as we work with scale invariant functions, or equivalently express all our statements about compositions in terms of ratios, we shall be subcompositionally coherent.

S2.2 Spurious correlation

Here we illustrate the phenomenon that Pearson [3] named "spurious correlation" by simulating three statistically independent mRNAs

$$\begin{aligned} \text{mRNA}_1 &\sim N(10, 1) \\ \text{mRNA}_2 &\sim N(10, 1) \\ \text{mRNA}_3 &\sim N(30, 3) \end{aligned}$$

and showing that the ratios $\text{mRNA}_1/\text{mRNA}_3$ and $\text{mRNA}_2/\text{mRNA}_3$ are correlated by virtue of their common divisor:

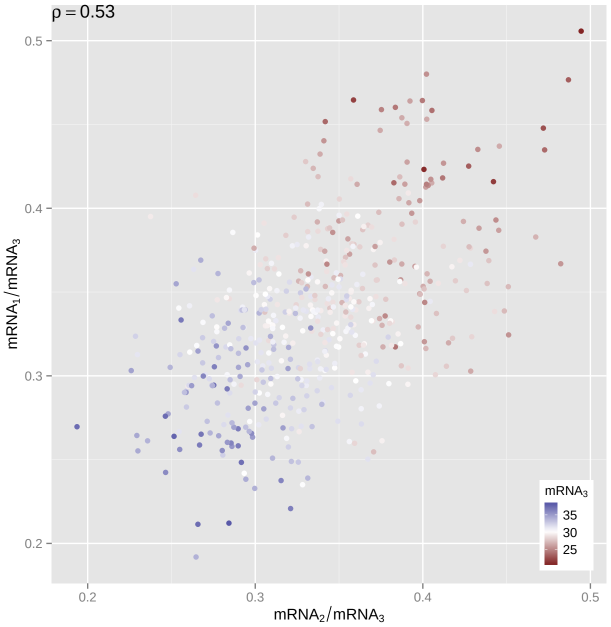

Figure S1: An illustration of the concept of spurious correlation (Pearson, 1897). Even though mRNA_1, mRNA_2 and mRNA_3 are statistically independent with sample correlations near zero, the ratios $\text{mRNA}_1/\text{mRNA}_3$ and $\text{mRNA}_2/\text{mRNA}_3$ have a correlation of 0.53 by virtue of their common divisor.

S2.3 Correlations between relative abundances tell us absolutely nothing

Here is a geometric illustration of why, in the absence of any other information or assumptions, correlations between relative values tell us nothing about relationships between the absolute values from which they were derived. We stress *in the absence of any other information or assumptions* to highlight an assumption that underpins many gene expression studies: that the total level of gene expression (i.e., absolute abundance of all kinds of mRNA) remains fairly constant across all experimental conditions. If this assumption holds, and all the mRNAs comprising that total are considered, the relative abundance of each kind of mRNA will be proportional to its absolute abundance, and analyses of correlation or “differential expression” of the relative values have clear interpretations. Our understanding is that the assumption of constant gene expression is often implicit and seldom tested; the revisitation of this assumption [24] should raise alarm bells about the inferences drawn from many gene expression studies.

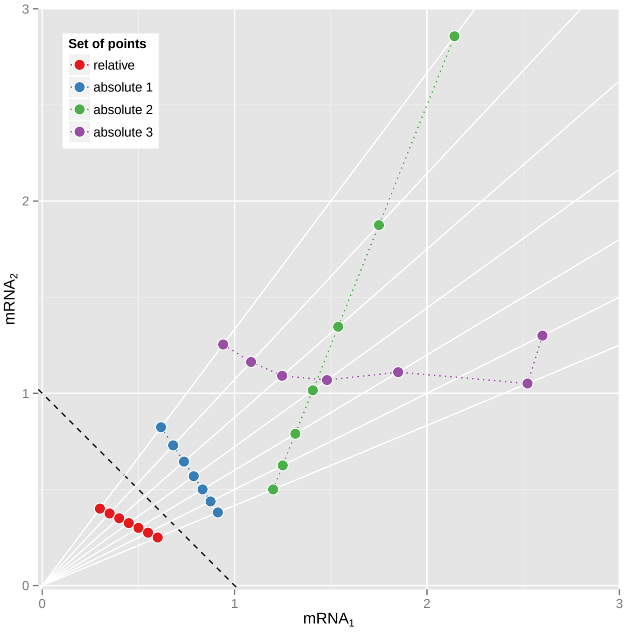

Figure S2: A geometric illustration of why correlation between relative abundances tells us nothing about the relationship between the absolute abundances that gave rise to them. A set of relative abundance pairs $(\text{mRNA}_1/\text{total}, \text{mRNA}_2/\text{total})$ is shown in red. The rays from origin through these points show the possible corresponding sets of absolute abundances. Thus, the perfectly negatively correlated relative abundances could have come from the blue, green or purple sets of points, whose Pearson correlations are -1, $+1$ and 0.0 respectively.

Figure S2 plots pairs of relative abundances $(\text{mRNA}_1/\text{total}, \text{mRNA}_2/\text{total})$ in red. For illustration, the relative abundances of the two different mRNAs are perfectly negatively correlated. What does this tell us about the relationship between the absolute abundances of mRNA_1 and mRNA_2? In the absence of any other information or assumptions: nothing. The red relative abundances could have come from absolute abundances that are perfectly negatively correlated (blue points), perfectly *positively* correlated (green points) or anywhere in between (purple points). Note that this is the case for both Pearson and Spearman correlation coefficients, and also the LP-Spearman coefficient proposed by Mukhopadhyay and Parzen [25]:

```
##          set  pearson spearman LP.spearman
## 1   relative -1.00000  -1.0000     -1.0000
## 2 absolute 1 -1.00000  -1.0000     -1.0000
## 3 absolute 2  1.00000   1.0000      1.0000
```

```
## 4 absolute 3  0.01256  -0.1429     -0.1429
```

Only when the relative abundances appear in proportion can we say something about the absolute abundances that gave rise to them, namely, that they too are proportional to one another:

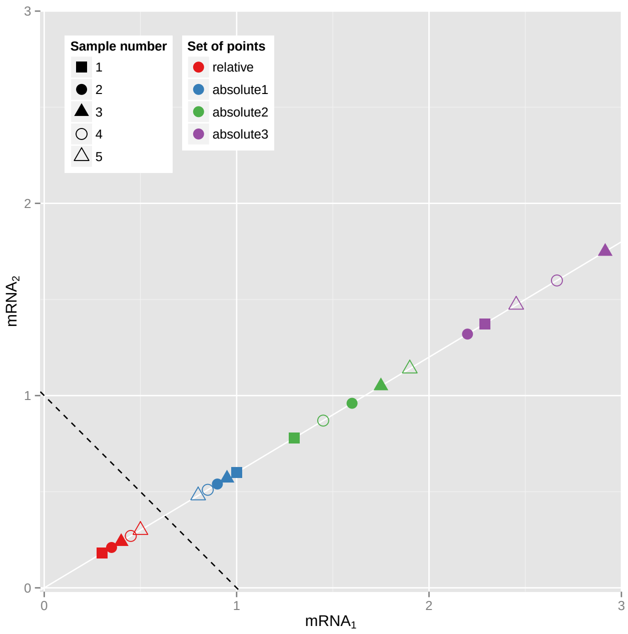

Figure S3: A geometric illustration of why pairs of relative abundances that are proportional must come from absolute abundances that are similarly proportional. A set of relative abundance pairs $(\mathrm{mRNA}_1/\mathrm{total}, \mathrm{mRNA}_2/\mathrm{total})$ is shown in red. The ray from origin through these points shows possible corresponding sets of absolute abundances. The blue, green or purple sets of point pairs have the same proportional relationship as the pairs of relative abundances, though not necessarily the same order or spread along the line of proportionality.

S2.4 Getting a sense of correlation, proportionality and logarithms

This section aims to illustrate some aspects of proportionality, correlation and logarithms that are important in understanding our paper and to put results on correlations between *logarithms* of measurements into perspective.

We focus on Pearson's correlation because it can be thought of as a measure of the extent to which two variables are linearly related, i.e., how well they fit the equation

$$y = mx + c$$

where m is the slope of the line of y plotted against x, and c is the y-intercept of that line, for example:

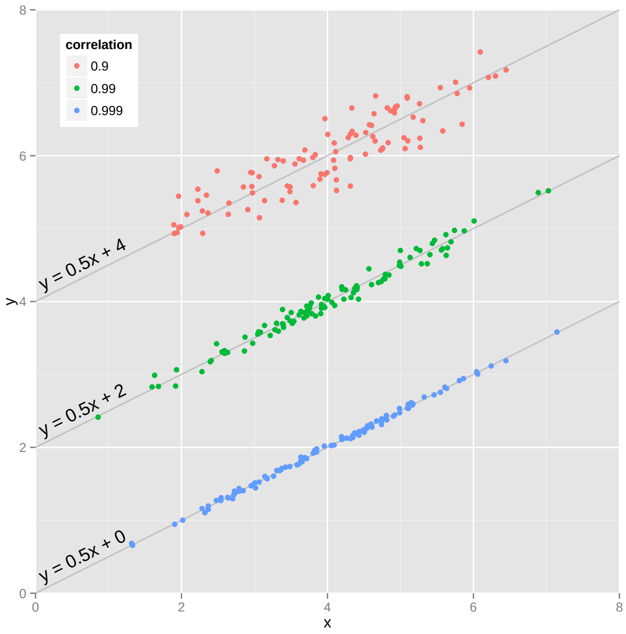

Figure S4: Three data sets with Pearson correlations of 0.999, 0.99, and 0.9, that fit lines with slopes of 0.5, and intercepts 0, 2 and 4, respectively.

Proportionality is stricter than correlation because it refers to the extent to which two variables fit the equation

$$y = mx,$$

that is, a line that passes through the origin. All three of the data sets in Figure S4 show pairs of values that are strongly (positively) correlated, but only the blue data are strongly proportional as well.

Here are examples of strongly proportional data on both natural, and log-scaled axes to illustrate that proportional pairs of variables lie on lines of slope 1 (i.e., 45°) when plotted on log-log axes:

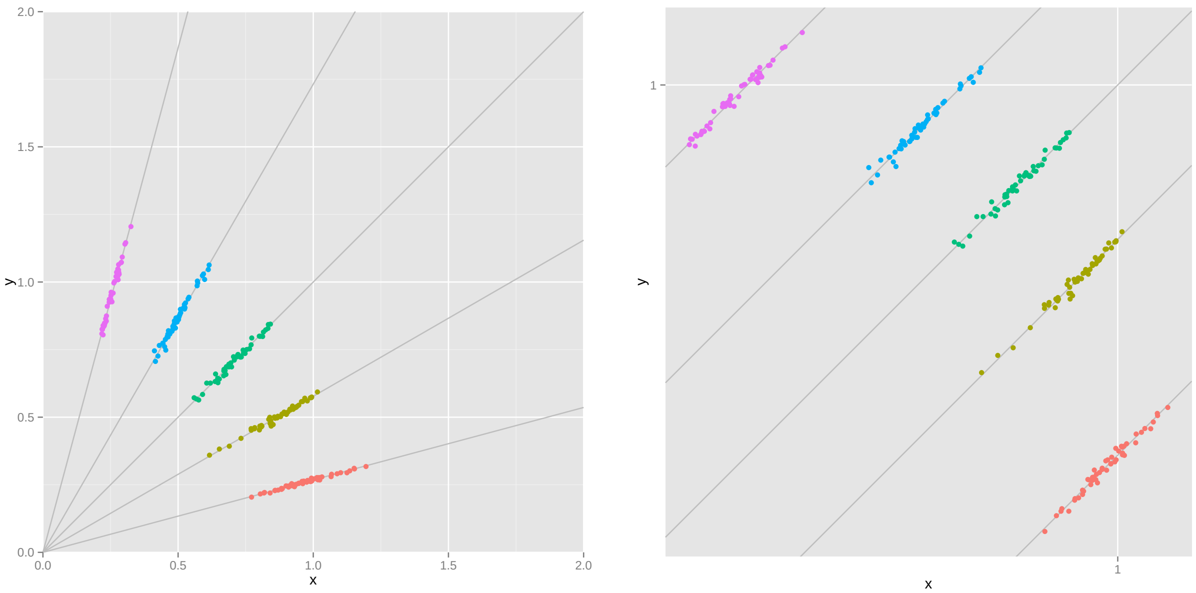

Figure S5: Five data sets whose pairs of values are strongly proportional plotted on natural (left) and logarithmically-scaled axes (right).

Postive data—relative and absolute—are often logarithmically transformed in molecular bioscience prior to analysis. Leaving aside the issue of how to analyse relative abundance data for a moment, we want to highlight the need for care in interpreting correlations in logarithmically transformed data. If, after taking logs, we find a strong linear relationship between $\log x$ and $\log y$ (giving us a Pearson correlation coefficient near $+1$ or -1) the interpretation of that relationship depends on the slope of the log-transformed pairs of datapoints:

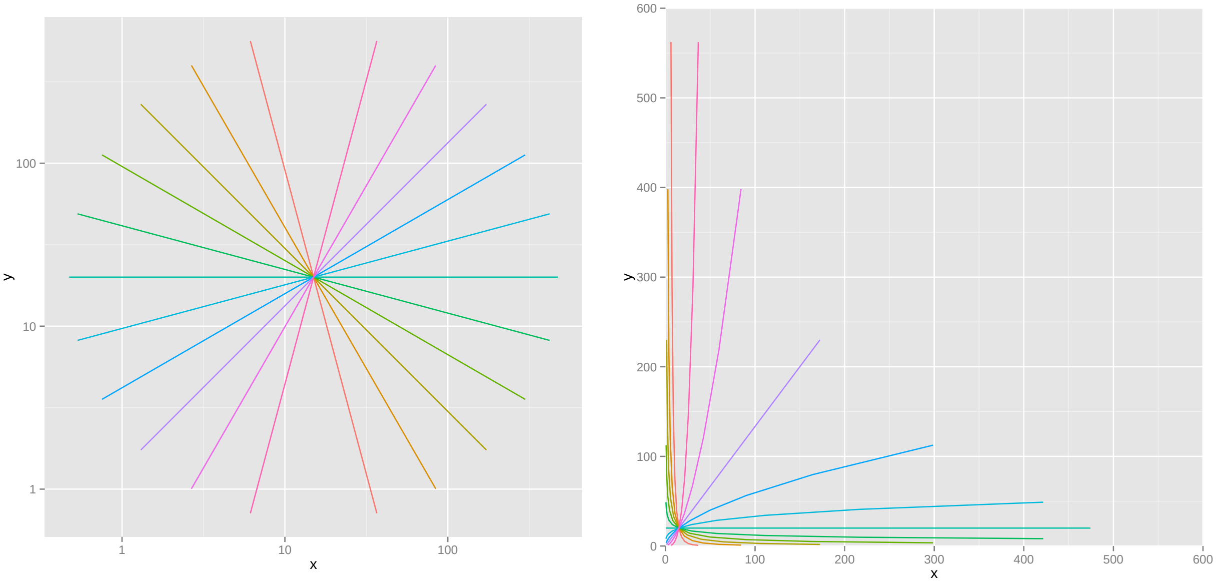

Figure S6: Lines of different slopes on a log-log scale (left) correspond to a variety of exponential curves on the natural scale (right).

While we see many molecular bioscience papers that mention correlations between different pairs of log-transformed measurements, we see far fewer that explore the interpretation of these relationships. We suspect that some readers will not appreciate that highly non-linear relationships can be implied on the original scale of measurement. We note too that correlations between log-transformed measurements imply a multiplicative error model and refer readers to [26] for discussion about the pros and cons of this assumption.

S2.5 Differential dilemma: when absolute and relative abundances change in different directions

Here are five different scenarios that can arise when considering "differential expression" with both relative and absolute abundances. Imagine that

- We can count the number of mRNA molecules present in a cell at a given point in time
- We can determine mRNA type, i.e., which gene an mRNA molecule was transcribed from.

Now imagine that

- We count and type the mRNAs from a cell undergoing Treatment A
- We do the same for a cell undergoing Treatment B
- We gather these counts for many such cells.

Finally

- We plot the counts of a particular type of mRNA in each cell under each Treatment
- We plot the proportions of that type of mRNA in each cell under each Treatment.

In which of the following scenarios would you say that the mRNA was differentially expressed?

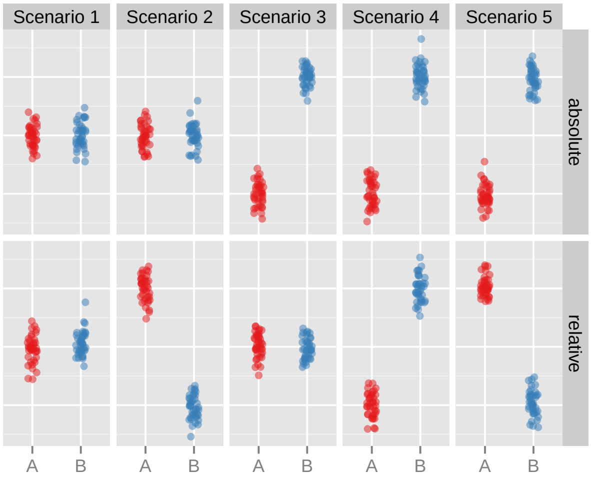

Figure S7: If we consider both absolute and relative abundances of an mRNA under two treatments, these five scenarios can arise (modulo treatment labels). Since it is quite possible for the absolute abundance of an mRNA to increase while its relative abundance decreases, we argue that the term "differential expression" needs careful qualification to avoid being misleading.

There is definitely something different going on in Scenarios 2-5, but what this figure should emphasise is that, with relative data, terms such as "over/under expression" and "up/down regulation" need to be carefully qualified to avoid misinterpretation.

S3 Preparing the data for analysis

We work with the following data sets from Marguerat et al. [3]:

1. RNA-seq measurement of yeast mRNA relative abundance at time 0
2. Microarray measurement of yeast mRNA abundances at 15 subsequent time points, relative to their abundances at time 0. (The yeast cells entered quiescence after time 0.)

S3.1 Downloading and extracting the data

Before importing the data into `R`, we

1. Downloaded Tables S1-S18 (XLSX 7.24 MB) to `./data`
2. Used Excel to save the RNA-seq measurements at time 0 (worksheet `Table_S2`) as comma-seperated values to `./data/RNA.seq.csv`
3. Used Excel to save the microarray measurements of the quiescence timecourse (worksheet `Table_15`) as comma-seperated values to `./data/microarray.csv`
4. Downloaded `Complex_annotation` to `./data`.

S3.2 Reading the data into `R`

Now we have the `.csv` versions, we bring them into `R` as follows:

```
RNA.seq           <- read.csv("./data/RNA.seq.csv",        header=T, skip=1)
microarray        <- read.csv("./data/microarray.csv",     header=T, skip=3)
names(microarray) <- sub("T", "timepoint", names(microarray))
go                <- read.csv("./data/Complex_annotation", header=T, sep="\t")
names(go)[6]      <- "Systematic.name"
```

S3.3 Creating a time course of absolute abundance

In the RNA-seq measurements at time 0, the counts observed for each mRNA should be roughly proportional to the absolute abundance of that mRNA in the yeast cells. We recognise that there are sample preparation issues and other factors that influence these counts, but a first order approximation will suffice for the points that this study seeks to make. For similar reasons, we will use only the complete (i.e., with `NA`s removed) `MM1` measurements throughout this analysis. (Studies whose focus is on understanding the biology of the system under study should, of course, use replicates sufficient to capture the variability of the system.)

```
# Average the sums all the copies-per-cell ("cpc") counts for MM1 and MM2,
# treating any NAs as 0
tmp <- data.frame(Systematic.name=RNA.seq$Systematic.name,
                  RNA.seq=rowSums(
                    RNA.seq[,grep("MM[12].*cpc.*", names(RNA.seq))],
                    na.rm=TRUE
                    )/2
                  )

# Drop any mRNAs that have a zero count in the RNA-seq
tmp <- subset(tmp, RNA.seq > 0)

# Do an inner join of Abs and the microarray data based on the Systematic names
tmp <- merge(tmp, microarray, by="Systematic.name")

# Now use the relative abundances at each microarray timepoint to multiply
# the initial mRNA copies per cell. Remove any rows that contain NAs
multipliers   <- as.matrix(tmp[, grep("timepoint", names(tmp))])
Abs           <- data.frame(tmp$RNA.seq * multipliers)
rownames(Abs) <- tmp[,"Systematic.name"]
Abs           <- na.omit(Abs)
Abs.t         <- as.data.frame(t(Abs))
```

We have now transformed 7289 RNA-seq observations, and 7054 observations into two dataframes of complete data: `Abs` (3031 genes at 16 timepoints) and its transpose `Abs.t`, containing the absolute abundances.

S3.4 Creating the corresponding time course of relative abundances

We create the time course of relative abundances by dividing the elements of each column by the column's total:

```
Rel   <- sweep(Abs,2,colSums(Abs, na.rm=TRUE),"/")
Rel.t <- as.data.frame(t(Rel))
```

S4 Problems with analyses that ignore the relative nature of data

S4.1 Correlations of relative abundances are misleading

Correlation—Pearson, Spearman or other—is the *bête noire* of compositional data, well known (in some circles) to lead to meaningless conclusions if applied to relative abundances. Here we calculate the correlation coefficients of all pairs of mRNAs (giving a 3031 $\times$ 3031 correlation matrix) for both absolute and relative abundances.

(Note that we are only calculating the correlations of the relative abundances to show how misleading they are! Don't do this, *unless* you happen to know the total absolute abundance is constant across all experimental conditions, in which case relative abundance is just a re-scaled version of absolute abundance throughout.)

```
Abs.cor <- stats::cor(Abs.t, use="pairwise.complete.obs")
Rel.cor <- stats::cor(Rel.t, use="pairwise.complete.obs")
```

The reason we invoke `stats::cor()` explicitly is that `cor()` is masked by the `compositions` package.

S4.1.1 Examining discrepancies in correlations I

Let's find the biggest discrepancies between the correlation matrices of absolute values, and of relative values:

```
Dif.cor     <- Abs.cor - Rel.cor
Dif.cor.max <- rownames(which(Dif.cor==max(Dif.cor), arr.ind=TRUE ))
Dif.cor.min <- rownames(which(Dif.cor==min(Dif.cor), arr.ind=TRUE ))
c(Dif.cor.max, max(Dif.cor))

## [1] "SPBC21C3.01c"     "SPAC823.06"       "1.95446837523687"

c(Dif.cor.min, min(Dif.cor))

## [1] "SPNCRNA.994"       "SPAC823.16c"       "-1.72032658783404"

rm(Dif.cor)
```

Having found the mRNAs whose correlations over the absolute and relative abundance timecourses are most different, we plot them. First, the two mRNAs with the largest positive difference between the correlation coefficient of their absolute values, and the correlation coefficient of their relative values:

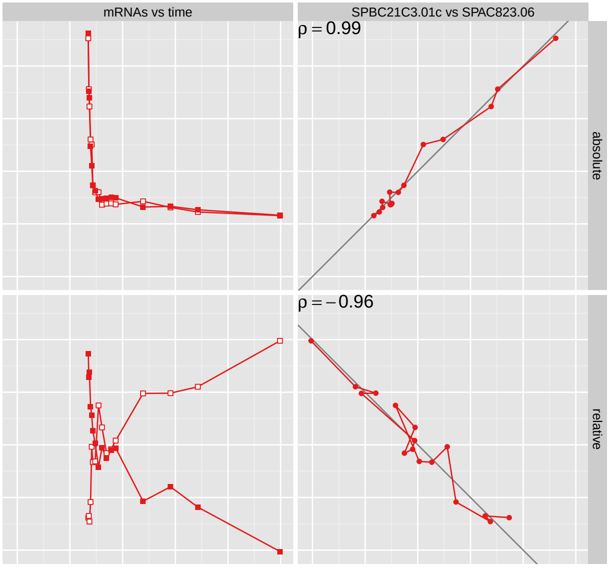

Figure S8: These four panels plot the abundances of SPBC21C3.01c and SPAC823.06. The upper panels refer to their absolute abundances; the lower panels to their relative abundances. The left panels show their abundances over time; the right panels plot their pairwise values. The right panels illustrate that correlation coeffiecient of the relative values is at the opposite extreme to that of the absolute values.

The top left panel of Figure S8 shows the absolute abundances of SPBC21C3.01c and SPAC823.06 over time, scaled and shifted so they can be plotted on the same graph. (Note that this scaling and shifting does not affect the correlation of these two time series.) The bottom left panel does the same for the *relative* abundances of these mRNAs. The right panels then plot the pairwise values of these two time series. These panels clearly show that the correlation coefficient of the two relative time series is at the opposite extreme to that of the two corresponding absolute series.

Here are the two mRNAs with the largest *negative* difference between the correlation coeffecient of their absolute values, and the correlation coefficient of their relative values:

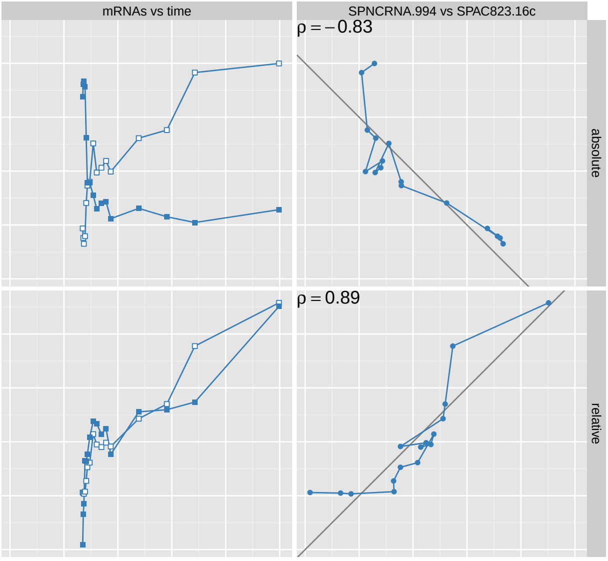

Figure S9: The pair of mRNAs labeled in blue in Figure S12, `SPNCRNA.994` and `SPAC823.16c`, shown on a linear scale. The upper panels show absolute abundances; the lower show relative abundances. The left panels show mRNA values over time; the right show the value of one mRNA plotted against the other at each timepoint. The correlation between the relative abundances is almost the complete opposite of that between the absolute abundances of this pair of mRNAs.

S4.1.2 Examining discrepancies in correlations II

Here we show how the apparent correlation between relative abundances depends on the components that are measured. This is the "subcompositional incoherence" problem discussed in Section S2.1.

Suppose we process the yeast samples so that the ten most abundant RNAs are removed on the grounds that they are *"taking up valuable sequencing capacity and resulting in a high signal-to-noise ratio that can make detection of the RNA species of interest difficult"* (a phrase used in describing Qiagen's GeneRead rRNA Depletion Kit discussed in [27]). First, let's find the ten RNA's that appear most abundant

```
Abs.total <- colSums(Abs.t)
top10 <- base::order(Abs.total, decreasing=TRUE)[1:10]
Abs.total[top10]

##   SPSNORNA.21  SPAC27E2.11c   SPNCRNA.906   SPSNORNA.20   SPAC1F8.07c
##          9248          7150          5306          4887          2985
##   SPBC19C2.07   SPBC26H8.01   SPAC4H3.10c SPAPB15E9.01c  SPCC13B11.01
##          2764          2055          1797          1688          1675
```

Now let's "deplete" these by setting their values to `NA`, then calculate the relative abundances in this depleted data, and their correlations

```
Depleted.Abs.t <- Abs.t
Depleted.Abs.t[,top10] <- NA
Depleted.Rel.t <- sweep(Depleted.Abs.t,1,rowSums(Depleted.Abs.t,na.rm=TRUE),"/")
Depleted.Rel.cor <- stats::cor(Depleted.Rel.t, use="pairwise.complete.obs")
```

Then, just as we did in the previous section, we look for the biggest discrepancies between the correlations of the relative abundances, and the correlations of the depleted relative abundances:

```
Dif.cor     <- Rel.cor - Depleted.Rel.cor
Dif.cor.max <- rownames(which(Dif.cor==max(Dif.cor, na.rm=TRUE), arr.ind=TRUE ))
Dif.cor.min <- rownames(which(Dif.cor==min(Dif.cor, na.rm=TRUE), arr.ind=TRUE ))
c(Dif.cor.max, max(Dif.cor, na.rm=TRUE))

## [1] "SPBC902.02c"       "SPAC1486.05"       "0.817572651002856"

c(Dif.cor.min, min(Dif.cor, na.rm=TRUE))

## [1] "SPAC9.03c"          "SPAC17G6.12"        "-0.752657145474333"

rm(Dif.cor)
```

In summary

- The apparent correlation of `SPBC902.02c` and `SPAC1486.05` in the "undepleted" relative abundances is 0.35, compared to -0.47 in the depleted relative abundances.
- The apparent correlation of `SPAC9.03c` and `SPAC17G6.12` in the "undepleted" relative abundances is -0.46, compared to 0.29 in the depleted relative abundances.

Changing the molecules (i.e., components) considered in the analysis of relative abundance changes the apparent correlations between molecules; the correlations are artefacts of the analysis approach rather than indications of statistical associations between the components of the system under study.

S4.2 Compare all correlation coefficients of the absolute and relative abundances

Pick a pair of mRNAs, say the first (`SPAC1002.02`) and second (`SPAC1002.03c`). The correlation of this pair's absolute values over the time course is 0.9824 while the correlation of their relative values is −0.3609. We could plot (0.9824, −0.3609) on a scatterplot and do the same for all other pairs of mRNAs were it not for the fact that there are 4.592 million such pairs — there would be a lot of overplotting. Instead, the following plot shows *counts* of the pairs binned on a 200 × 200 grid, and we annotate the extremes found in the previous section:

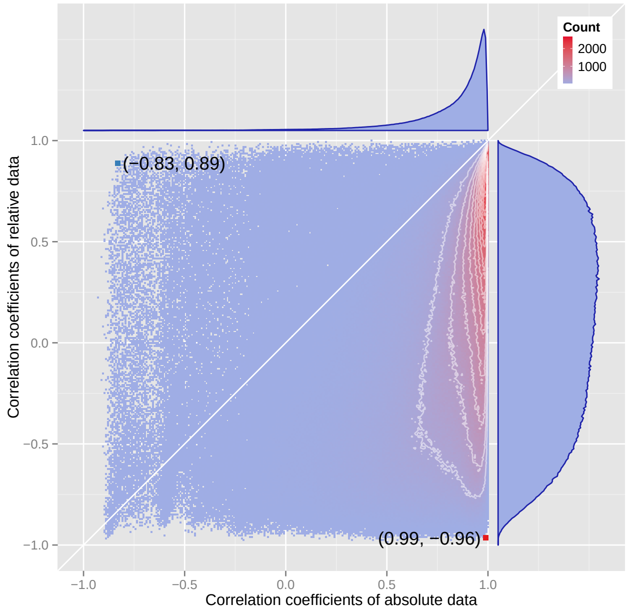

Figure S10: a 2D histogram of the sample correlation coefficient observed for the relative abundances of a given pair of mRNAs, against the correlation coefficient observed for the absolute abundances of that same pair, over all pairs. The red and blue points correspond to the red and blue pairs of mRNA in Figure S12. White contour lines are shown at intervals of 100 counts. The top marginal histogram shows that the absolute abundances of most pairs are very strongly correlated. The right marginal histogram shows "the negative bias difficulty" [28, Section 3.3] of closure on correlation—here, correlations between relative abundances bear no relationship to the corresponding correlations between absolute abundances.

We can do the same summary plot for the correlations of the “undepleted” and depleted relative abundances:

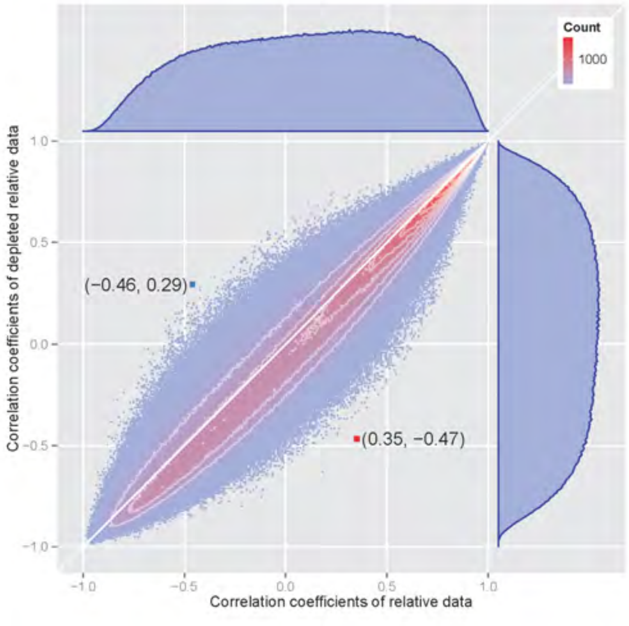

Figure S11: A 2D histogram of the correlation coefficient observed for the relative abundances of a given pair of mRNAs in a sample where the ten most abundant mRNAs have been removed, against the correlation coefficient observed for the relative abundances of that same pair, over all pairs. White contour lines are shown at intervals of 100 counts. While the distribution of the correlation coefficient pairs lies more on the diagonal than in the preceding figure, it is clear that correlation of relative abundances is sensitive to what is in (or out of) the total, i.e., correlation is *not* subcompositionally coherent.

The removal of the top ten most abundant mRNAs affects the apparent correlations between the relative abundances of the other mRNAs. This emphasises the fact that the relative abundances of different mRNAs are not independent of one another. If the relative abundance of one mRNA increases, the relative abundances of some other mRNAs must *decrease*, and *vice versa*. Consequently the apparent correlation between relative abundances depends on which components are considered to make up the sample under study.

In short, if you deplete the most abundant mRNAs from the sample and use correlation to measure association between relative abundances, you get different correlations than if you had left those mRNAs in. Correlations of relative abundances cannot be relied upon to make coherent inferences about the relationships between pairs of genes.

S4.3 Plot the time series

Here is the data in all its glory, in two plots of absolute and relative values, overlaid with the mRNA pairs with the biggest discrepancies between the correlation matrix of absolute values, and the correlation matrix of relative values.

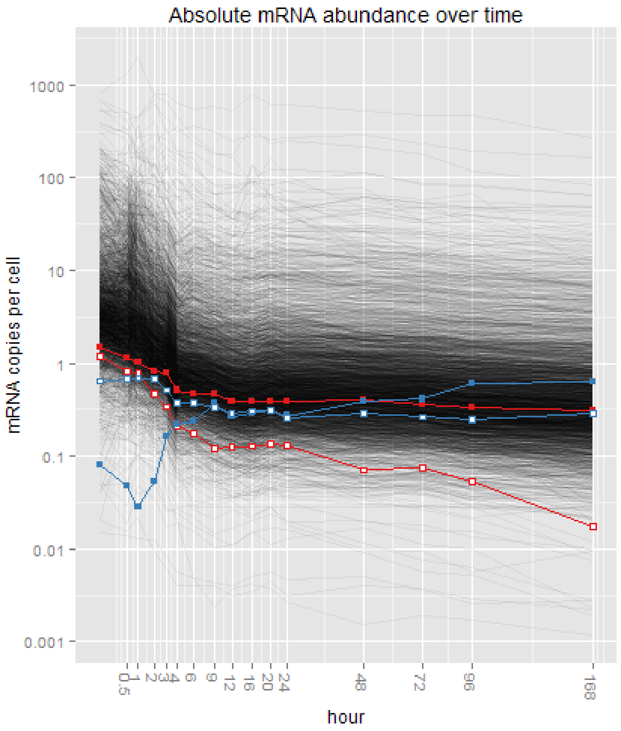

Figure S12: Absolute abundances of 3031 yeast messenger RNAs over the 16-point time course from [3]. The y-axis is scaled logarithmically and the x-axis is on a square-root scale so that all the data can be clearly seen. Each grey line corresponds to the expression levels of a particular mRNA. The red and blue pairs of mRNAs correspond to those analysed in Section S4.1.1.

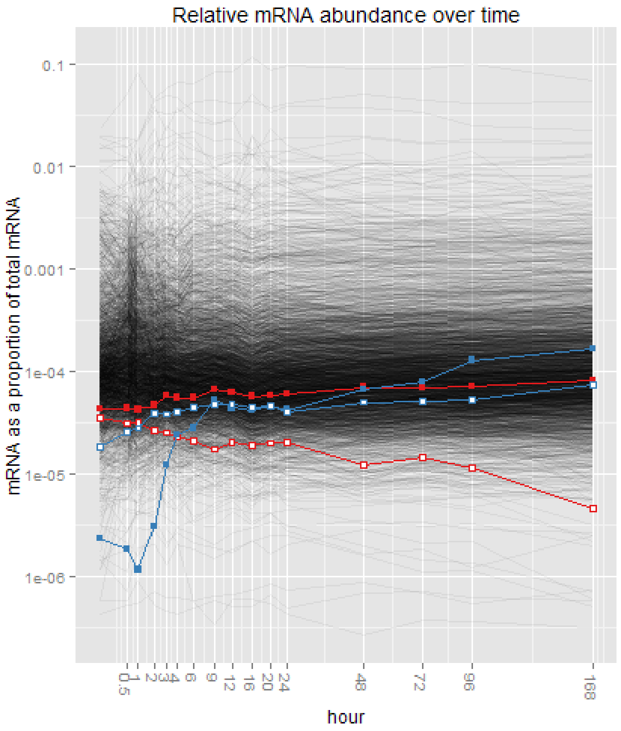

Figure S13: Relative abundances of 3031 yeast messenger RNAs over the 16-point time course from [3]. The y-axis is scaled logarithmically and the x-axis is on a square-root scale so that all the data can be clearly seen. Each grey line corresponds to the expression levels of a particular mRNA. The red and blue pairs of mRNAs correspond to those analysed in Section S4.1.1.

S4.4 Challenges in interpreting "differential expression" with the yeast data

Univariate statistical tests for differential expression have been popular in the analysis of relative abundances in bioscience. Much effort has been invested in developing approaches to deal with small numbers of observations and large numbers of tests. Until recently, comparatively little attention has been given to "...the commonly believed, though rarely stated, assumption that the absolute amount of total mRNA in each cell is similar across different cell types or experimental perturbations" [24].

When absolute total mRNA varies, the relationship between the relative and absolute abundance of a component is perhaps most easily understood in terms of fold change over time. If we write the fold change in amount (absolute abundance) of mRNA_i from time t_1 to time t_2 as

$$\text{fc}_{\text{abs}}(i) = \frac{\text{amount of mRNA}_i \text{ at time } t_2}{\text{amount of mRNA}_i \text{ at time } t_1}$$

then the apparent fold change in *relative* abundance is

$$\begin{aligned} \text{fc}_{\text{rel}}(i) &= \frac{\text{amount of mRNA}_i \text{ at time } t_2}{\text{total amount of mRNA at time } t_2} \cdot \frac{\text{total amount of mRNA at time } t_1}{\text{amount of mRNA}_i \text{ at time } t_1} \\ &= \text{fc}_{\text{abs}}(i)/\text{fc}_{\text{abs}}(\text{total}). \end{aligned} \tag{1}$$

When the total absolute abundance of mRNA stays constant over time $\text{fc}_{\text{abs}}(\text{total}) = 1$ and the fold changes in both absolute and relative abundance of mRNA_i are equal: $\text{fc}_{\text{abs}}(i) = \text{fc}_{\text{rel}}(i)$. When the total absolute abundance of mRNA varies, fold changes in absolute and relative abundances of each mRNA are no longer equal and can change in *different* directions.

Let's look at how the fold changes of absolute abundances and the fold changes of relative abundances create challenges with interpretation. Problems arise when the total absolute abundance of all the components changes over time. This is certainly the case for the yeast mRNA data, and we highlight timepoints 0 and 3 for further attention later:

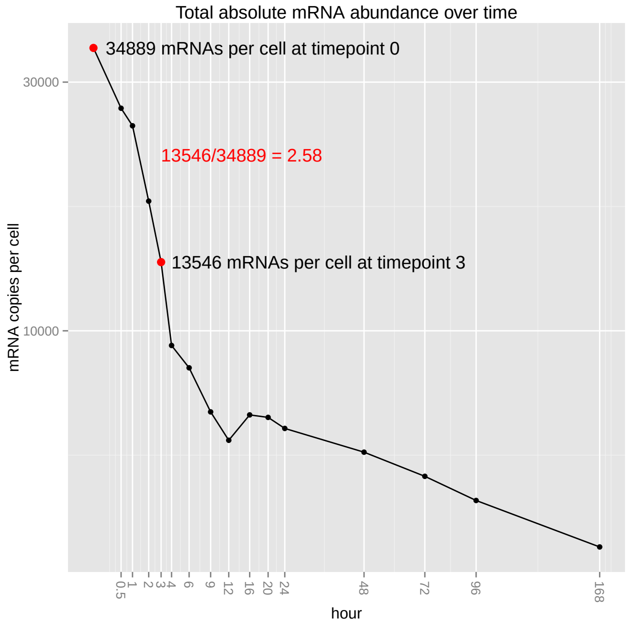

Figure S14: Total abundance of yeast mRNAs in copies per cell over the 16-point time course. Times 0 and 3 are highlighted for further study.

Consider the fold changes in relative and absolute abundances that occur for each of the 3031 mRNAs from timepoint 0 to timepoint 3. We do this in two ways, first by plotting the fold change of the relative data against the fold change of the absolute data (on a log-log scale):

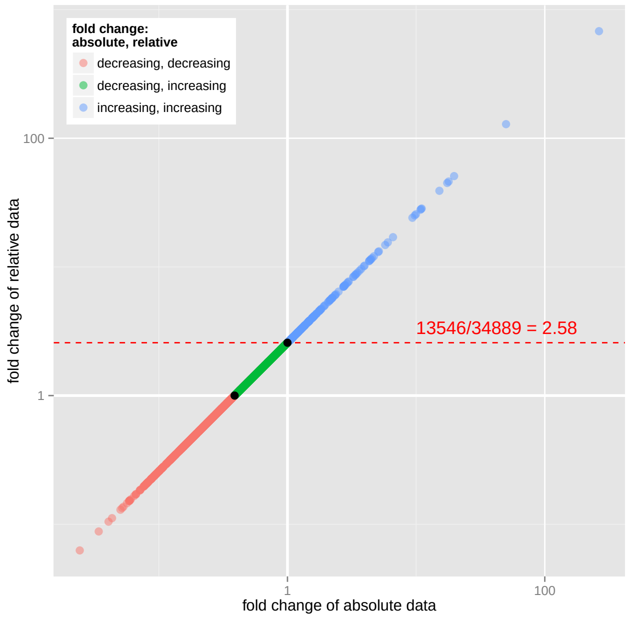

Figure S15: The fold change of the relative abundance of an mRNA from time 0 to time 3 plotted against the fold change of its *absolute* abundance, for all mRNAs. These data are plotted on a log-log scale.

Second, to emphasise that the relative fold changes are the same as the absolute fold changes multiplied by a constant, we show the histograms of the absolute and the relative fold changes. It turns out that, from time 0 to time 3, there are 1399 mRNAs whose absolute abundance decreases, but whose relative abundance *increases*:

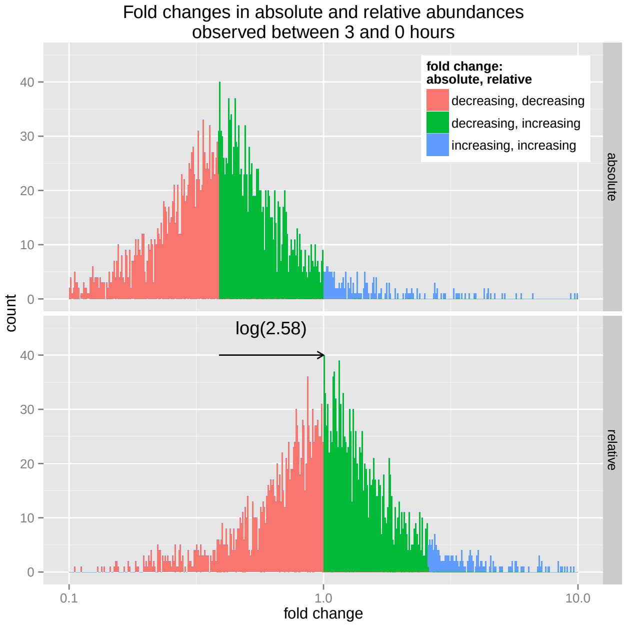

Figure S16: Histograms of fold changes in absolute abundance (top panel) and fold changes in relative abundance (bottom panel) of the yeast mRNAs between 0 and 3 hours. The colours indicate mRNAs whose absolute and relative fold changes are both decreasing (red), decreasing and increasing (green), and both increasing (blue). The x-axis is on a log scale and the shift of log(2.56) relates to the ratio of total mRNA abundances at 0 and 3 hours (Figure S14). By Equation 1, the distribution of fold changes in *relative* abundance is the same as that for *absolute* abundance shifted right by $\log(\mathrm{fc}_{\mathrm{abs}}(\mathrm{total})) = \log(2.56)$.

S5 Measuring association as "goodness of fit to proportionality"

S5.1 Visualising pairs of variables with different slope and fit

We have shown how logratio variance can be factored into two terms

$$\mathrm{Var}\log(x/y) = \mathrm{Var}(\log x)(1 + \beta^2 - 2\beta|r|)$$

where

- β is the slope of the Standardised Major Axis (SMA) [29]
- r is the correlation coefficient of $\log x$ and $\log y$

To help give you a sense of what data look like with different β, r values, here are scatter plots of data with different slopes (ranging from $\pm 60°$) and r^2 values from 1 to 0. In each panel we print the corresponding value of

$$\phi(\log x, \log y) = 1 + \beta^2 - 2\beta|r|$$

in red.

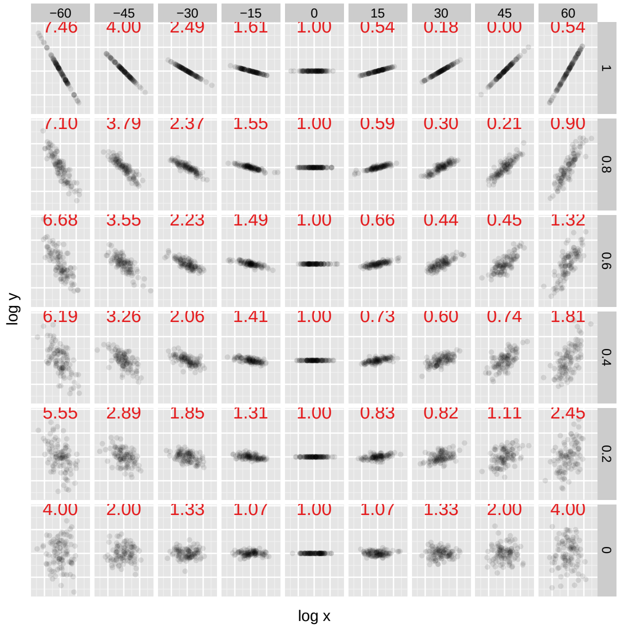

Figure S17: Each panel plots 100 points sampled from a bivariate lognormal distribution with different slopes (β ranging from $\pm 60°$) and coefficients of determination (r^2 ranging from 1 to 0). The corresponding value of $\phi(\log x, \log y)$ is printed in red.

S5.2 Plotting proportionality against slope and fit

Building on the plot of the previous section, here is a coloured contour plot of $\phi()$ as a function of slope β and coefficient of determination r^2—the white dot markes the minimum (0):

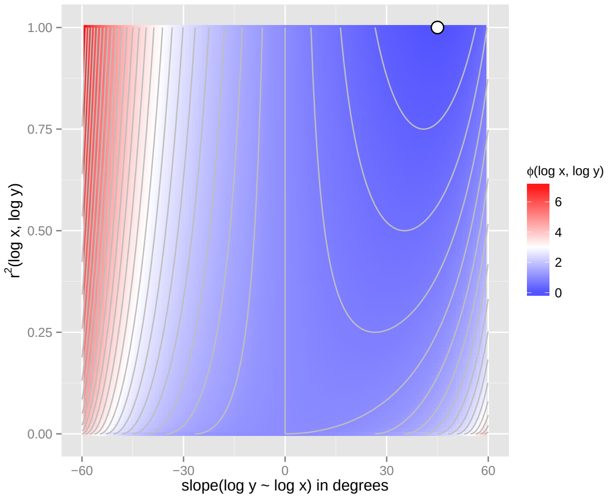

Figure S18: $\phi(\log x, \log y)$ as a function of the slope and coefficient of determination of the standard major axis of $\log y$ versus $\log x$. The grey lines show the contours of $\phi(\log x, \log y)$ in increments of 0.25. The hollow dot shows the minimum of $\phi(\log x, \log y)$ attained at a slope of $\beta = 1$ (i.e., 45°) and $r^2 = 1$.

S5.3 Properties of "goodness of fit to proportionality"

Clearly $\phi(\log x, \log y) \geq 0$ and can be thought of as a measure of dissimilarity ("disproportionality") between components x and y, achieving 0 when x and y are perfectly proportional. However, $\phi()$ does not satisfy the properties of a *d*istance—most obviously, it is not symmetric unless $\beta = 1$:

$$\begin{aligned}\phi(\log x, \log y) &= 1 + \beta^2 - 2\beta|r| \\ \phi(\log y, \log x) &= 1 + \frac{1}{\beta^2} - 2\frac{1}{\beta}|r|.\end{aligned}$$

We could symmetrise $\phi()$ by averaging $\phi(\log x, \log y)$ and $\phi(\log y, \log x)$, as is done for Kullback-Liebler divergence. Or we could take the maximum, or minimum of the two terms. We note also that we could define a new and symmetric distance function in terms of β and r, e.g.,

$$|\log \beta| + \log 2 - \log(r + 1)$$

In this paper, we are most interested in pairs of variables where β and r are near 1 and want to preserve the link between $\phi(\log x, \log y)$, β and r. Hence, our approach to the symmetrisation of $\phi()$ is simply to work with $\phi(\log x_i, \log x_j)$ where $i < j$, in effect, the lower triangle of the matrix of ϕ values between all pairs of components.

S5.4 Calculating "goodness of fit to proportionality" for the yeast data

In this section we calculate $\phi(\text{clr}(\text{Rel})_i, \text{clr}(\text{Rel})_j))$. Here clr() refers to the *centred logratio transformation* of each of the 16 mRNA compositions observed. The clr representation of composition $\mathbf{x} = (x_1, \ldots, x_i, \ldots, x_D)$ is the logarithm of the components after dividing by the geometric mean of $\mathbf{x}$:

$$\text{clr}(\mathbf{x}) = \left(\log \frac{x_1}{\text{g}_\text{m}(\mathbf{x})}, \ldots, \log \frac{x_i}{\text{g}_\text{m}(\mathbf{x})}, \ldots, \log \frac{x_D}{\text{g}_\text{m}(\mathbf{x})},\right)$$

ensuring that the sum of the elements of $\text{clr}(\mathbf{x})$ is zero.

This representation ensures that all linear operations on the transformed data will produce compositions and is known as "working in the simplex". This is important for hypothesis testing and also to ensure that the $\phi()$ values we calculate are on a consistent scale (as discussed later in Section S6):

```
Rel.clr     <- as.data.frame(clr(Rel.t))
```

We'll calculate $\phi()$ shortly, but before we do that, we need to prepare a dataframe so we can plot $\phi()$ in relation to the slopes and coefficients of determination (r^2) values of standardised major axis (SMA) fits. Most of the functions used in this analysis are tucked away in `yeast.functions.R`, but here we set out how the function `sma.df()` efficiently (i.e., vectorised for `R`) calculates the slopes of the SMA of all pairs of variables in a dataframe `df`, and the p-values of the hypothesis tests that those slopes are equal to 1.

Warton et al. [29, Table 1] describe the calculations as follows. We wish to estimate the line $Y = \alpha + \beta X$ from N pairs of observations of X and Y. The SMA estimate of slope is

$$\hat{\beta} = \text{sign}(s_{xy})\frac{s_y}{s_x}$$

where s_{xy} is the sample covariance of X and Y, and s_x^2 the sample variance of X. $\hat{\beta}$ is element `b` of the list returned by `sma.df()` below.

To test the hypothesis that the SMA slope is 1, Warton et al. [29] test whether $X + Y$ and $X - Y$ are uncorrelated. To vectorise this computation, we make use of the fact that

$$\rho(X + Y, X - Y) = \frac{(s_x^2 - s_y^2)^2}{(s_x^2 + s_y^2)^2 - 4s_{xy}^2}.$$

Now here's the `R` code to implement that:

```
# Perform sma fits on all pairs of columns in df
sma.df <- function(df){
  df.cor <- stats::cor(df, use="pairwise.complete.obs")
  df.var <- stats::cov(df, use="pairwise.complete.obs")
  df.sd  <- sqrt(diag(df.var))

  # Following the approach of Warton et al. Biol. Rev. (2006), 81, pp. 259-291
  # r.rf2 = cor(X+Y, X-Y)^2
  #       = (var(X) - var(Y))^2  /  ((var(X) + var(Y))^2 - 4cov(X,Y)^2)
  r.rf2   <-
    (outer(diag(df.var), diag(df.var), "-")^2 ) /
    (outer(diag(df.var), diag(df.var), "+")^2 - 4 * df.var^2 )

  # At this point the diagonal of r.rf2 will be 0/0 = NaN. The correlation should be 0
  diag(r.rf2) <- 0
  res.dof     <- nrow(df) - 2
  F           <- r.rf2/(1 - r.rf2) * res.dof

  list(b=sign(df.cor) * outer(df.sd, df.sd, "/"), # slope = sign(s_xy) s_y/s_x
```

```
        p=1 - pf(F, 1, res.dof),                    # p-value of the test that b = 1
        r2=df.cor^2)                                # the squared correlation coefficient
}
```

This vectorisation strategy means that `sma.df()` calculates all 4.5 million slopes, p-values and r^2 values for the yeast data in under a minute, compared with 40 minutes or so for the naïve implementation.

Note too that we only calculate the p-values to show later why hypothesis testing is not as useful as goodness-of-fit; in practice, you could speed up `sma.df()` further by dropping the p-value calculation all together.

```
Rel.sma     <- sma.df(Rel.clr)
```

We calculate $\phi(\mathrm{clr(Rel)}_i, \mathrm{clr(Rel)}_j)$ by making use of the relationship

$$\phi(\log X, \log Y) = \frac{\mathrm{var}(\log(X/Y))}{\mathrm{var}(\log X)}.$$

```
Rel.vlr     <- vlr(Rel) # The variance of the log-ratios (i.e., the variation array)
Rel.clr.var <- apply(Rel.clr, 2, var) # The variance of each variable
Rel.phi     <- sweep(Rel.vlr, 2, Rel.clr.var, FUN="/")
```

Now that we have $\phi(\mathrm{clr(Rel)}_i, \mathrm{clr(Rel)}_j))$, $\mathrm{Var}(\log(\mathrm{Rel}_i/\mathrm{Rel}_j))$, $\hat{\beta}$, r^2 and the relevant p-values, we've done most of our calculations and the next step rearranges the results so that we can plot them. At this point in the script, memory is running out though, so we have to resort to a few minor tricks.

```
# Find the indices of the lower triangle
lt                 <- which(col(Rel.sma$b)<row(Rel.sma$b),arr.ind=FALSE)
lt.ind             <- which(col(Rel.sma$b)<row(Rel.sma$b),arr.ind=TRUE)

# Find the row minimum of the lower triangle of phi
Rel.phi.min        <- lt.row.min(Rel.phi)

# At this stage in the script, 32-bit implmentations of R will struggle
# to get everything into memory in one hit.
# I find growing the dataframe incrementally helps, as does getting rid
# of any objects that aren't used from here on in
Rel.sma.df         <- data.frame(
  row=factor(rownames(Rel.sma$b)[lt.ind[,"row"]]),
  col=factor(colnames(Rel.sma$b)[lt.ind[,"col"]])
  )
Rel.sma.df$b       <- Rel.sma$b[lt]
Rel.sma.df$p       <- Rel.sma$p[lt]
Rel.sma.df$r2      <- Rel.sma$r2[lt]
Rel.sma.df$vlr     <- Rel.vlr[lt]
Rel.sma.df$phi     <- Rel.phi[lt]
Rel.sma.df$Abs.cor <- Abs.cor[lt]
```

S5.5 How does proportionality relate to the correlations of absolute abundances of yeast mRNAs?

Previously, we summarised the pairs of correlation coefficients of absolute and relative data in a 2D histogram. Here we use a similar strategy to show the joint distribution of correlation coeffecients of absolute data, and $\phi()$ values of *relative* data.

Remember, $\phi()$ measures the degree of proportionality between pairs of variables; the lower it is, the more the variables exhibit a proportional relationship. Remember also that pairs of variables can show strong correlations but low proportionality when they are linearly related, but with a non-zero intercept term (see Section S2.4).

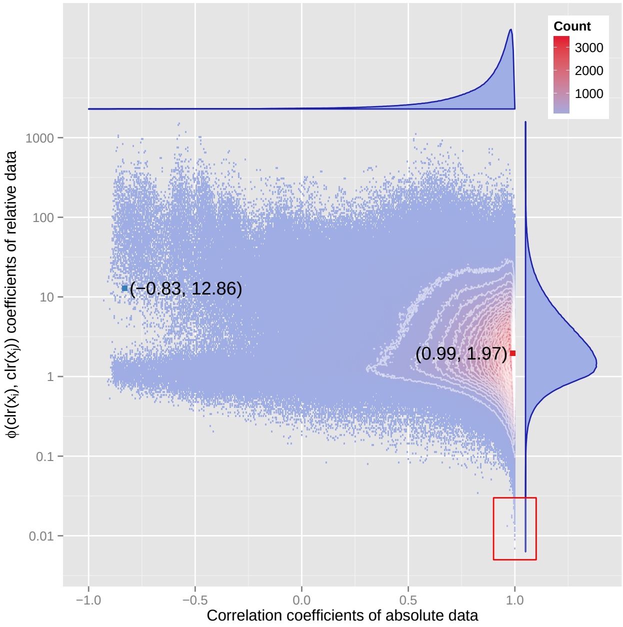

Figure S19: A 2D histogram of $\phi(\text{clr}(x_i), \text{clr}(x_j))$ for the relative abundances of a given pair (i, j) of mRNAs, against the correlation coefficient observed for the absolute abundances of that same pair, over all pairs. The red and blue points correspond to the red and blue pairs of mRNA in Figure S12. White contour lines are again shown at intervals of 100 counts and the top marginal histogram is the same as in the left-hand figure. The few mRNA pairs that are strongly proportional (within the red rectangle) are also strongly positively correlated. However, the converse is not true: strong positive correlation between mRNAs does not imply that they are strongly proportional.

S5.6 What slopes, fits, proportionalities and logratio variances are seen for yeast mRNA pairs?

Here is a 2D histogram of the slopes and r^2 values of each of the 4.592 million pairs of yeast mRNAs. The red rectangle shows an area that we will zoom in on.

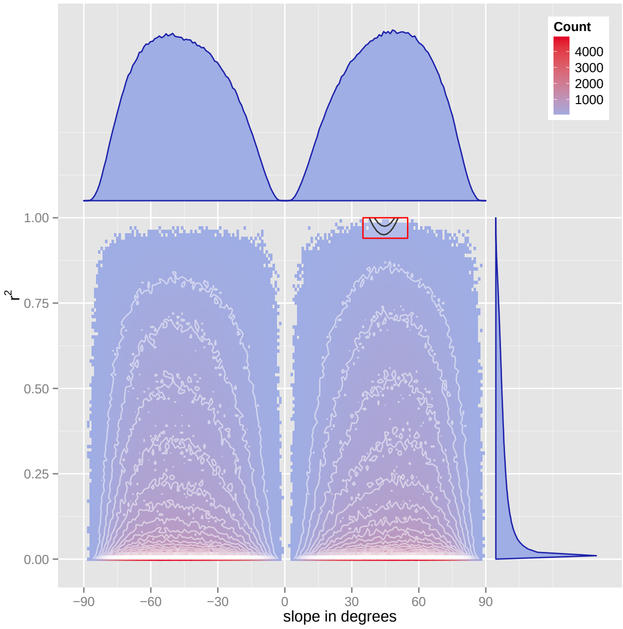

Figure S20: The bivariate distribution of slope and r^2 values observed in all $3031 \times 3030/2 \approx 4.5$ million pairs of mRNA relative abundances in our time course. The marginal histograms show the distribution of slope values (top) and r^2 values (right). White contour lines are spaced at intervals of 100.

Let's zoom in and, instead of plotting a 2D histogram, just do a scatterplot of the (β, r^2) pairs, coloured by their $\phi()$ value, with a couple of the contours of $\phi()$ thrown in for good measure:

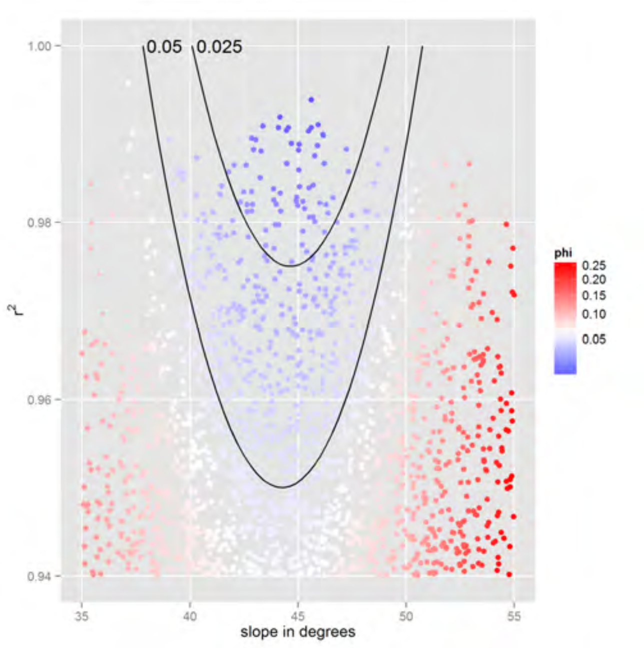

Figure S21: A zoomed in view of the area inside the red rectangle in the previous plot. The black lines show the 0.05 and 0.025 contours of $\phi(\text{clr}(x_i), \text{clr}(x_j))$ and points are coloured according to that statistic.

Now we pull back a little so we can see a lot more pairs

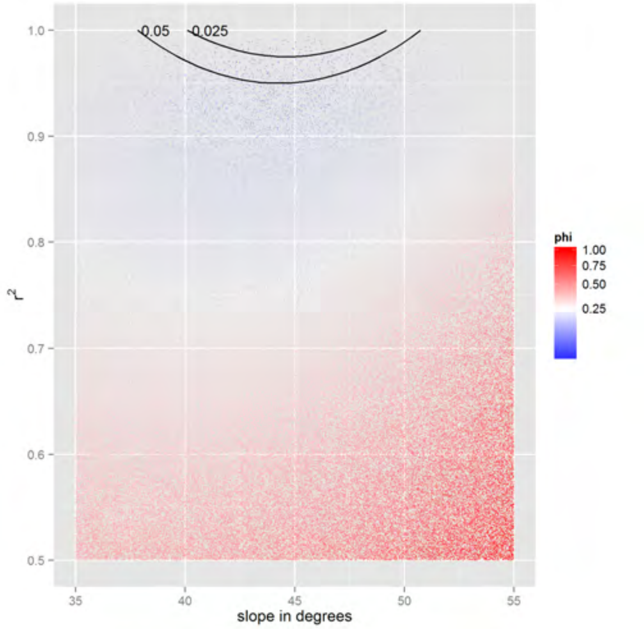

Figure S22: A view of slope and r^2 values encompassing more data than the previous figure, again with points coloured by $\phi(\text{clr}(x_i), \text{clr}(x_j))$.

Contrast the preceding distributions with the p-values of the hypothesis test that each pair is proportional:

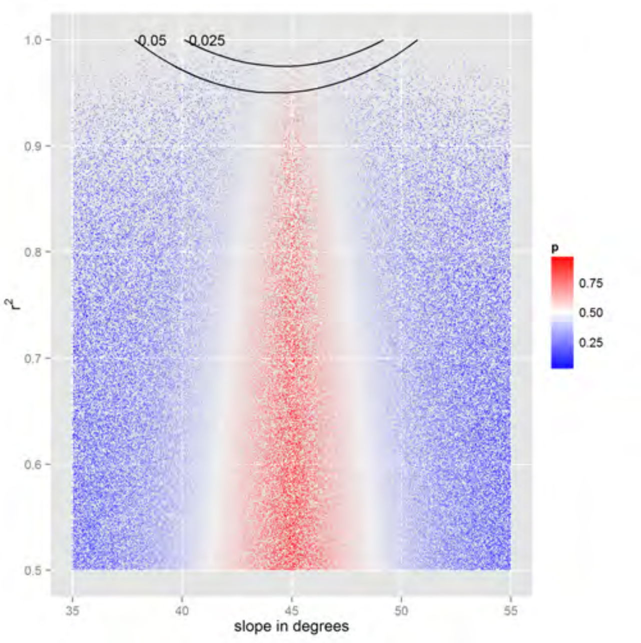

Figure S23: The same points as the previous figure coloured this time by the p-value of the slope test of isometry.

. . . in essence, any pair whose SMA slope is around 1 gets a high p-value, no matter the goodness of fit (i.e., the r^2). Since interest focuses on mRNAs that exhibit strong proportionality, this hypothesis testing approach is not as useful as a goodness-of-fit approach (more of which later in Section S5.8).

What about the variance of logratio as a measure of proportionality? Here we plot the (β, r^2) pairs, coloured by the variance of their logratio:

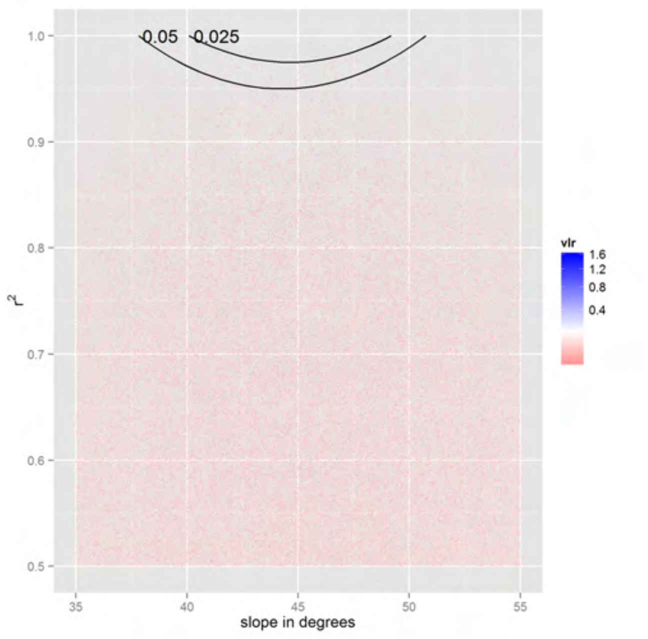

Figure S24: The same points as the previous figure coloured this time by $\mathrm{Var}(\log(x_i/x_j))$.

This illustrates what Friedman and Alm [30] pointed out about the variance of the logratio of variables that are not exactly proportional

> "it is hard to interpret as it lacks a scale. That is, it is unclear what constitutes a large or small value. . . (does a value of 0.1 indicate strong dependence, weak dependence, or no dependence?)"

S5.7 Are there "clumps" of proportional mRNAs?

Plotting the cumulative distribution of the minimum $\phi()$ value of each mRNA gives us a sense of how many of the mRNAs exhibit strong proportionality with some other mRNA

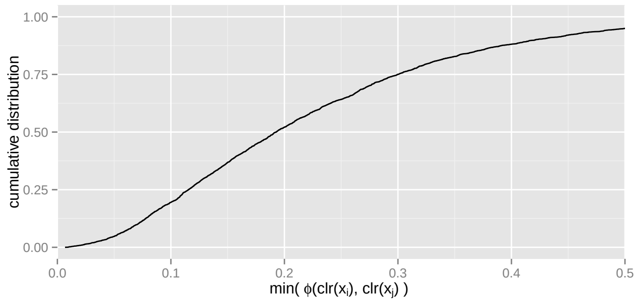

Figure S25: Cumulative distribution of the minimum $\phi()$ value of each mRNA.

Now we have the basis of an approach that can give us some strongly proportional pairs of mRNA. We select all the pairs with $\phi() < 0.05$. This gives us 145 mRNAs, about 5% of the data. We could have set a higher threshold of $\phi()$, say 0.1, but the object of this analysis is to illustrate how proportionality can be used as a measure of association for relative abundance data, and 145 mRNAs is enough to be illustrative without becoming unwieldy.

```
Rel.sma.lo.phi <- subset(Rel.sma.df, phi <  0.05)
```

Next we plot these mRNA pairs with low $\phi()$ values on the natural scale and on the log-log scale

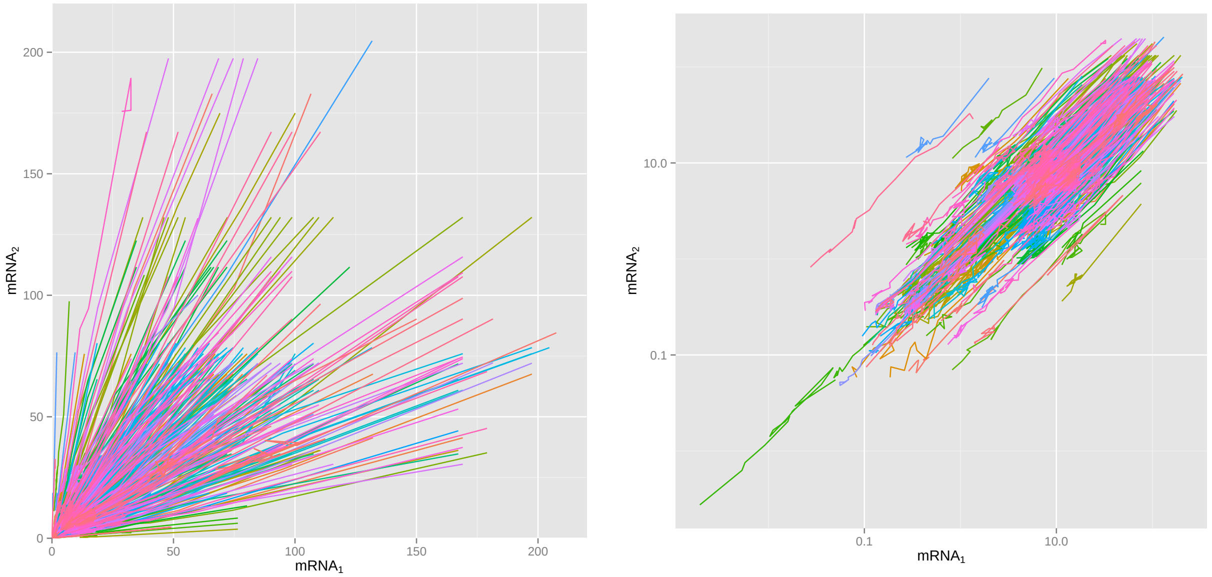

Figure S26: Absolute expression levels of the 424 pairs of mRNAs with $\phi(\text{clr}(x_i), \text{clr}(x_j)) < 0.05$ plotted on a natural scale (left) and on the log-log scale (right).

Both these plots show that low $\phi()$ values correspond to pairs of mRNAs that exhibit strong proportionality.

Note that instead of using $\phi(\text{clr}(x_i), \text{clr}(x_j)) < 0.05$ we could have selected a subset of strongly proportional mRNAs using some other criterion involving slope and correlation, e.g.,

$$r^2 > 5(\beta - 1)^2 + 0.8.$$

S5.8 Why not use a hypothesis testing approach?

What if we use the p-values instead to select pairs of mRNAs that are strongly proportional?

```
Rel.sma.hi.pval <- subset(Rel.sma.df, p >  0.9999)
```

Here are the mRNA pairs with high *p*-values plotted on the natural scale and log-log scale

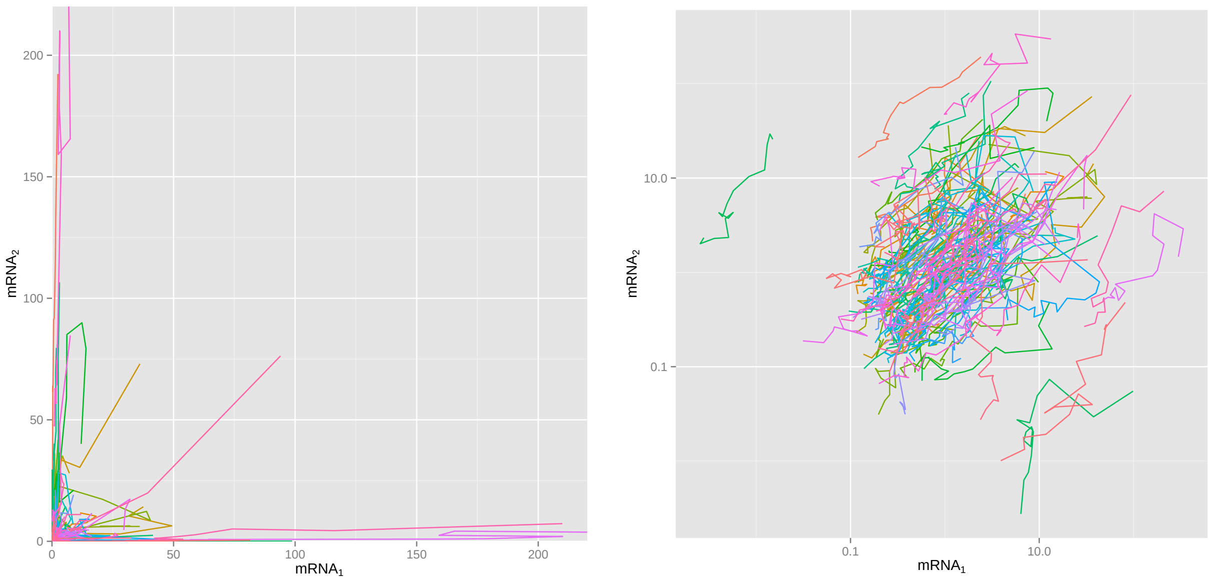

Figure S27: Absolute expression levels of the 136 pairs of mRNAs with slope test *p*-values> 0.9999 plotted on the natural scale (left) and log-log scale (right).

These two plots illustrate why we prefer to select proportional mRNAs on the basis of a goodness-of-fit statistic rather than *p*-values. In essence, goodness-of-fit gives us a way to *compare* the relationships between different pairs of components.

S5.9 Finding "clumps" of proportional variables

As a measure of association, we can use $\phi()$ as the basis of some familiar analyses, such as network visualisation. Here we lay out a graph in which the vertices represent mRNAs and the edges between them indicate strong proportionality across the time course:

```
Rel.sma.lo.phi <- subset(Rel.sma.df, phi <  0.05)
g              <- graph.data.frame(Rel.sma.lo.phi, directed=FALSE)
plot(
  g,
  layout=layout.fruchterman.reingold.grid(g, weight=0.05/E(g)$phi),
  vertex.size=1,
  vertex.color="black",
  vertex.label=NA
  )
```

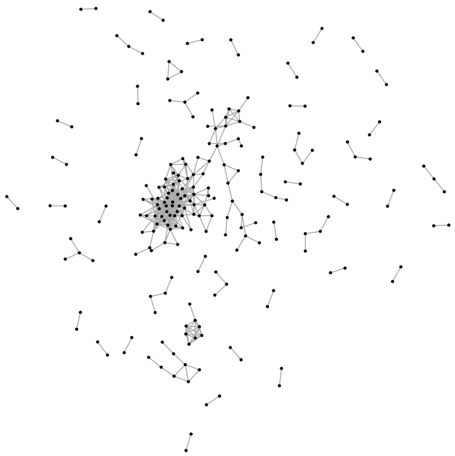

Figure S28: A graph of the proportionality relationships between the 424 pairs of mRNAs with $\phi(\mathrm{clr}(x_i), \mathrm{clr}(x_j)) < 0.05$.

Next, we retrieve additional information from Pombase about the mRNAs appearing in this graph.

```
g.clust     <- clusters(g)
g.df        <- data.frame(
  Systematic.name=V(g)$name,
  cluster=g.clust$membership,
  cluster.size=g.clust$csize[g.clust$membership]
  )
```

Unfortunately, we have to do this next step manually by pasting the string produced by

```
cat(as.character(g.df$mRNA), sep=", ")
```

into `http://www.pombase.org/spombe/query` with the `Systematic IDs` filter selected, then saving the results into `data/proportional mRNAs.tsv`. Then we read that file back in and merge it with `g.df`:

```
pombase.df <- read.csv("data/proportional mRNAs.tsv", sep="\t")
nrow(pombase.df)

## [1] 217

nrow(g.df)

## [1] 218

g.df <- merge(g.df, pombase.df, by.x="Systematic.name", by.y="ensembl_id", all=TRUE)
write.csv(g.df, "data/proportional mRNAs.csv")
saveRDS(g.df, "RDS/g.df.RDS")

## Warning:  cannot open compressed file 'RDS/g.df.RDS', probable reason 'No such file or directory'
## Error:  cannot open the connection

rm(pombase.df)
```

After noting that there were 218 vertices in the graph (`length(V(g)` = 218) but only 217 rows in `pombase.df` we saw that one of our mRNAs was missing:

```
subset(g.df, is.na(name))

##     Systematic.name cluster cluster.size name chromosome description
## 209    SPNCRNA.1291      15            4 <NA>       <NA>        <NA>
##     feature_type strand start end
## 209         <NA>     NA    NA  NA
```

and a search of `http://www.pombase.org/status/new-and-removed-genes` revealed that `SPNCRNA.1291` was merged with `SPNCRNA.519` on 2011-12-16. For the purposes of this study it is easiest just to copy the details of `SPNCRNA.519` into the `SPNCRNA.1291` row of `g.df`:

```
g.df[g.df$Systematic.name=="SPNCRNA.1291",-(1:3)] <-
  g.df[g.df$Systematic.name=="SPNCRNA.519",-(1:3)]
```

See Section S7 for tabulation of all the mRNAs in `g.df` along with functional information.

Let's get the names of the mRNAs in the largest connected cluster in that graph—we'll use these in the next section:

```
g.max       <- induced.subgraph(
  g, which(g.clust$membership %in% which(g.clust$csize == max(g.clust$csize)))
  )
g.max.names <- V(g.max)$name
```

Let's also have a look at a couple of smaller clusters

```
g.8       <- induced.subgraph(
  g, which(g.clust$membership %in% which(g.clust$csize == 8))
  )
g.8.names <- V(g.8)$name
plot(
  g.8,
  layout=layout.fruchterman.reingold.grid(g.8, weight=0.05/E(g.8)$phi),
  vertex.size=3,
  vertex.color="white"
  )
```

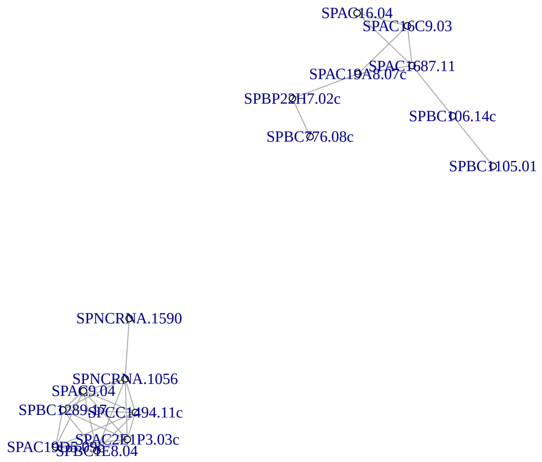

S5.10 Visualising proportionality with a heatmap

$\phi()$ can also be used as the basis for a clustered heatmap. Before doing that, we must make the matrix of $\phi(\log x, \log y)$ values symmetric so it can serve as a distance matrix:

```
# This next line symmetrises Rel.phi. In effect, it copies the lower triangle
# of Rel.phi onto the upper triangle
Rel.phi.sym <- as.matrix(as.dist(Rel.phi))
Rel.phi.hc  <- hclust(as.dist(Rel.phi))
plot.heat(Rel.phi.sym, Rel.phi.hc)
rm(Rel.phi.hc)
```

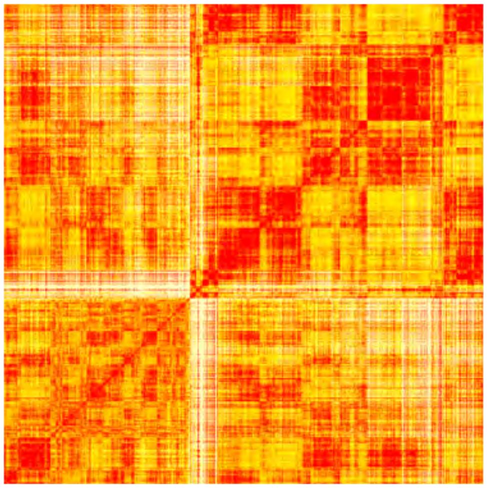

Figure S29: Heatmap of symmetrised $\phi()$ matrix.

Even though there is structure evident, that's far too many mRNA's to make sense of. On the next page we look at a more manageable subset.

S5.11 Visualising clusters of proportional mRNAs with a heatmap

This next plot shows a heatmap for the 96 mRNAs in the largest connected cluster of the graph in Figure S28. Table S4 in Section S7 shows that this cluster ($c = 3$) of strongly proportional mRNAs relates to the ribosome.

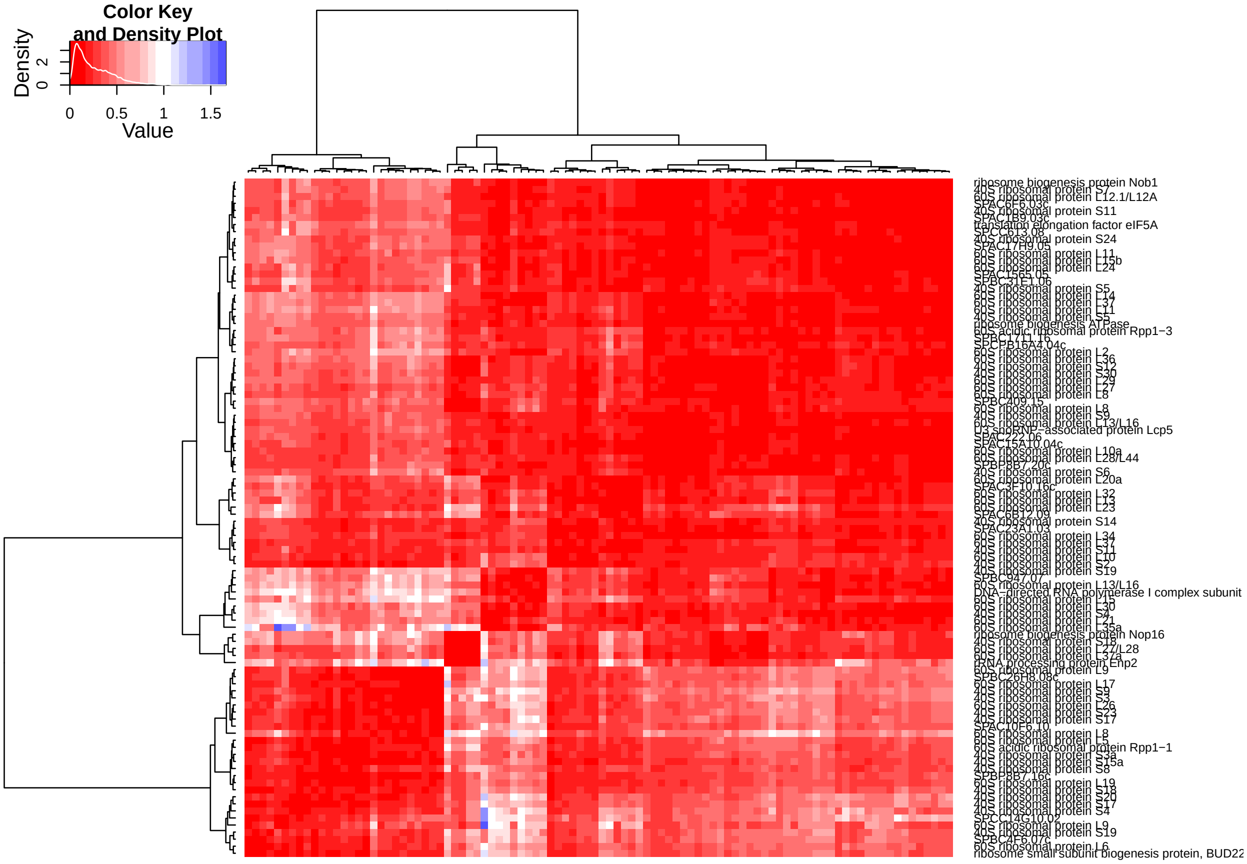

Figure S30: Heatmap visualisation of the 96 mRNA cluster seen in Figure S28.

It is remarkable how strongly proportional these mRNAs are to one another within this group. Here are their relative abundances over time, with a blue line showing the geometric mean

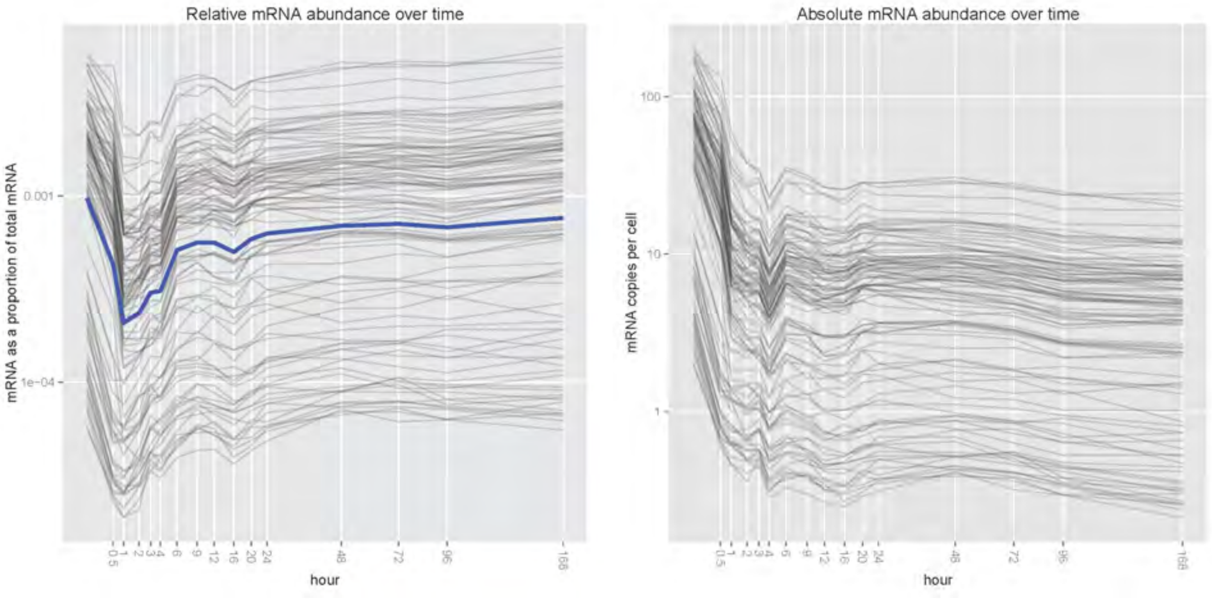

Figure S31: (Left) The relative abundances of each of the mRNAs from the 96 mRNA cluster seen in Figure S28 over time. The geometric mean at each timepoint is shown in blue. (Right) The corresponding absolute abundances for reference.

Now we plot the values of each mRNA as multiples of the geometric mean expression level at each time point. This shows that the mRNA expression levels within this group stay pretty well locked in fixed ratios, raising interesting questions as to the molecular mechanisms that ensure this, and the extent to which this will be the case in other situations.

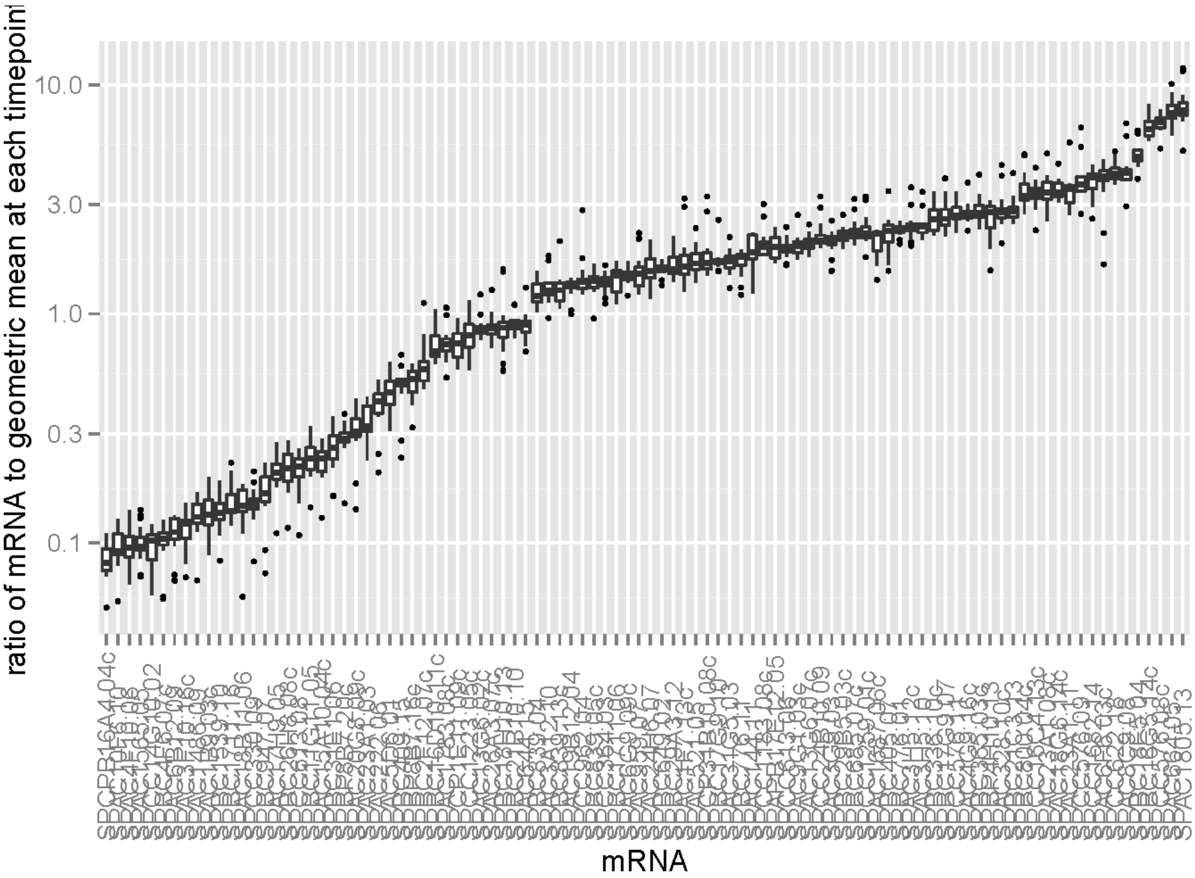

Figure S32: Each of the mRNAs from the 96 mRNA cluster seen in Figure S28 divided by the geometric mean of the mRNAs at each timepoint

Now let's show how $\phi()$ can be used for hierarchical clustering when there are several clusters present. To do that, we extract the rows and columns of the `Rel.phi.sym` matrix that contain values of $\phi() < 0.025$, build a clustered heatmap, and use the clustering to cut the matrix into 6 groups.

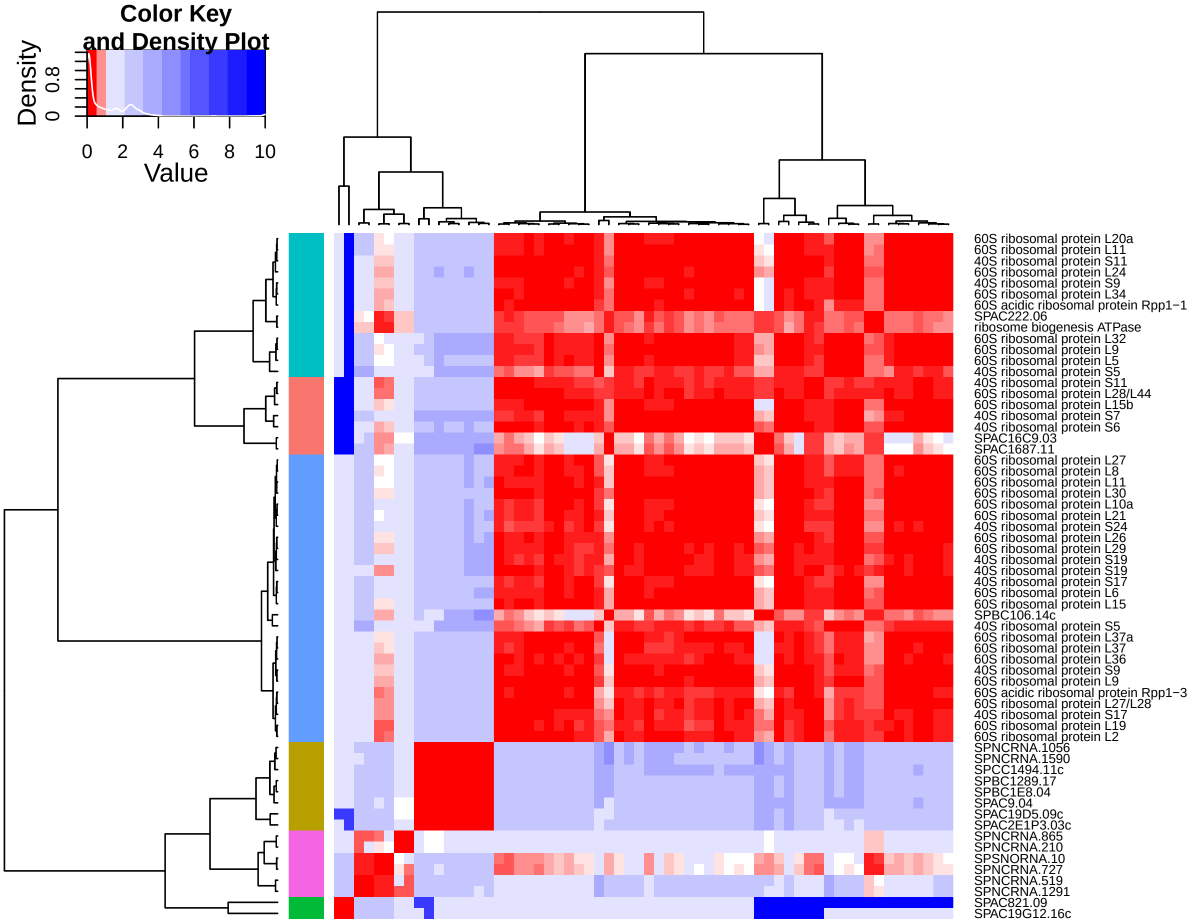

Figure S33: Heatmap visualisation of the 66 pairs of mRNAs with $\phi(\text{clr}(x_i), \text{clr}(x_j)) < 0.025$. The hierarchical clustering of these components is cut into six colour-coded groups, shown at the left edge of the heatmap.

Let's look at the abundances of the mRNAs in these six clusters over time

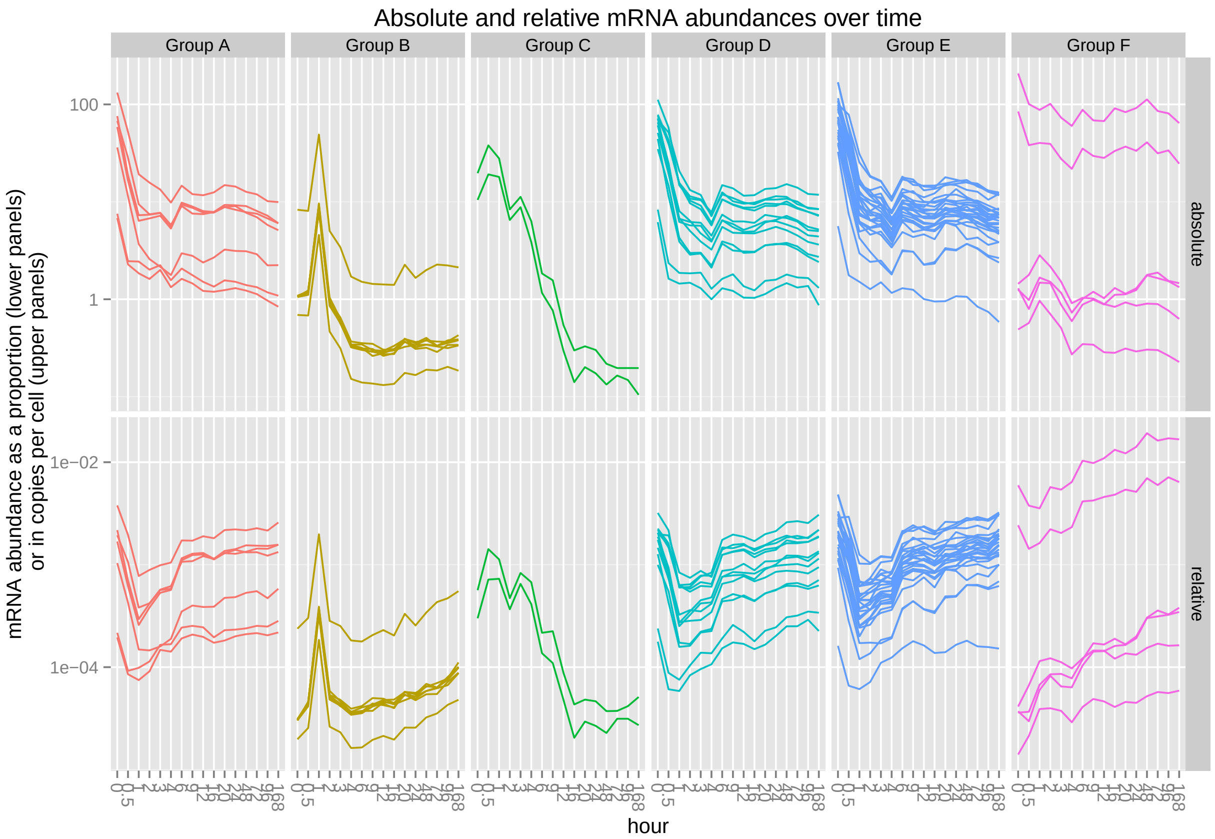

Figure S34: Absolute and relative abundances of the 66 pairs of mRNAs clustered into six groups in Figure S33. The line colours correspond to the colour-coding of groups in Figure S33.

Let's apply this approach to look at two of the smaller clusters in Figure S28:

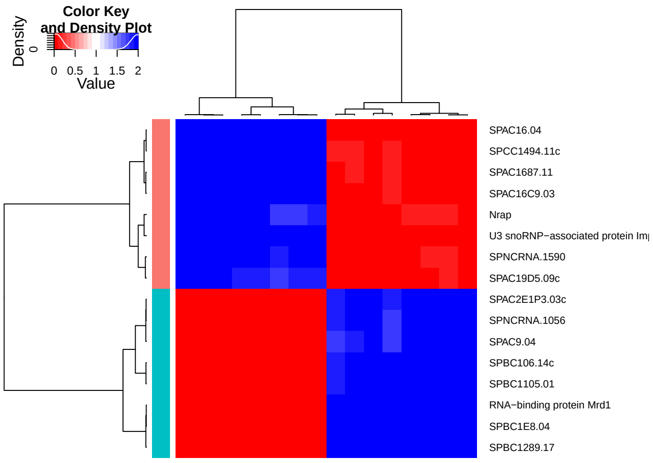

Figure S35: Heatmap visualisation of two smaller mRNA clusters seen in Figure S28.

Here are the abundances of the mRNAs in these two clusters over time

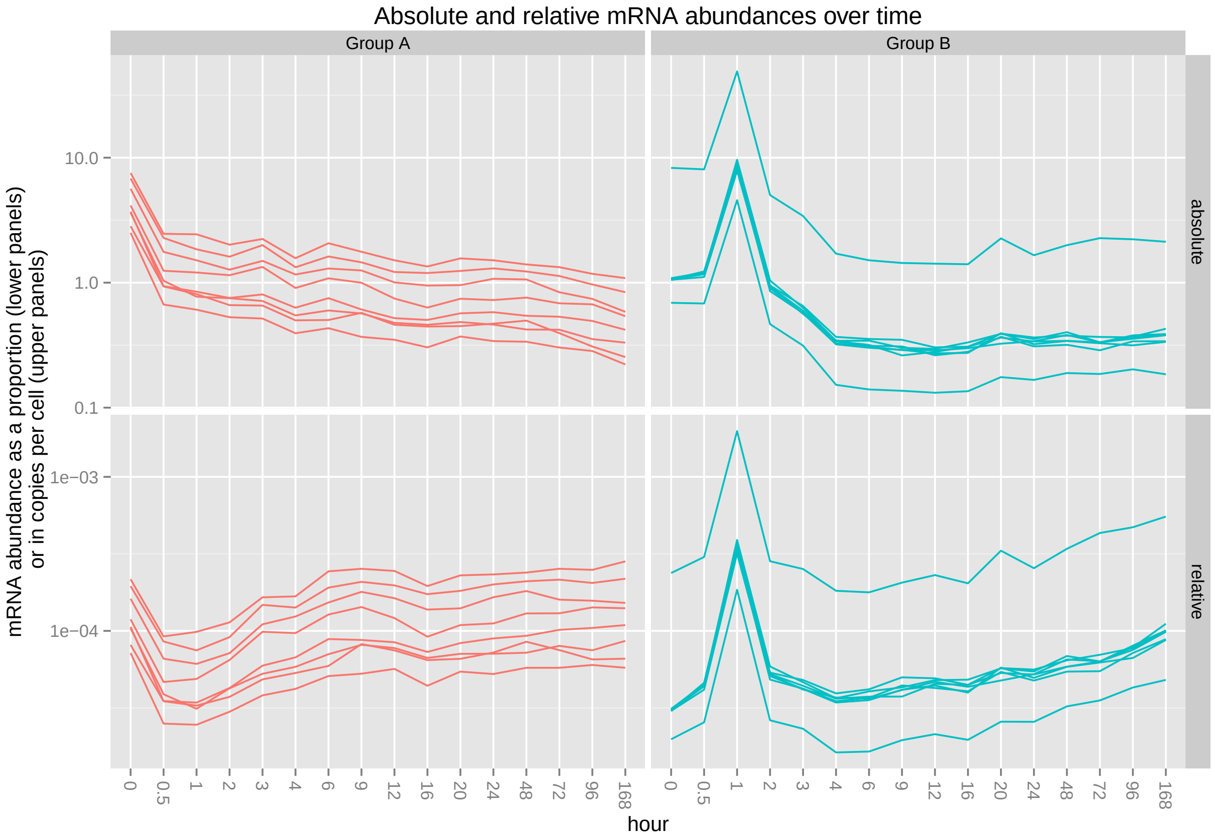

Figure S36: Absolute and relative abundances of the 16 pairs of mRNAs clustered into 2 groups in Figure S35. The line colours correspond to the colour-coding of groups in Figure S35.

Let's look closer at the genes in Group A (which corresponds to cluster 7 in Table S4)

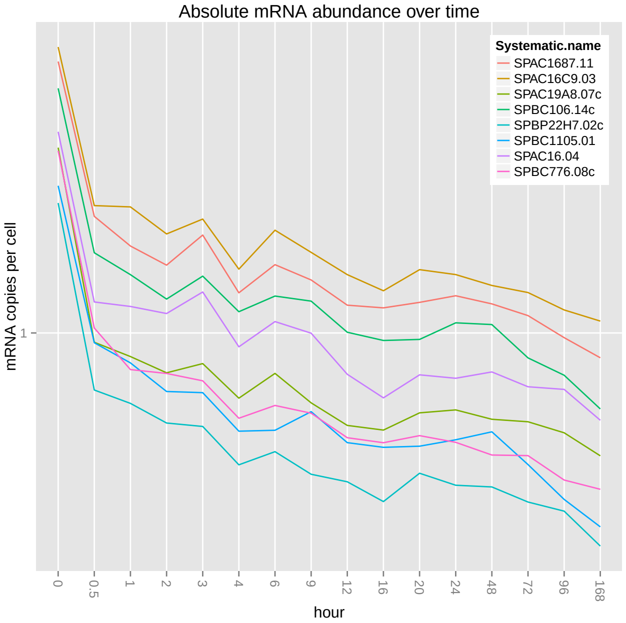

Figure S37: Absolute abundances of the 8 mRNAs in Group A.

c	Systematic ID	Description	Feature type	Chr	Str
7	SPAC16.04	tRNA dihydrouridine synthase Dus3 (predicted)	protein_coding	I	1
7	SPAC1687.11	rRNA methyltransferase Spb1 (predicted)	protein_coding	I	1
7	SPAC16C9.03	export adaptor Nmd3 (predicted)	protein_coding	I	1
7	SPAC19A8.07c	U3 snoRNP-associated protein Imp4 (predicted)	protein_coding	I	1
7	SPBC106.14c	SDA1 family protein (predicted)	protein_coding	II	-1
7	SPBC1105.01	rRNA processing protein Rrp12-like (predicted)	protein_coding	II	1
7	SPBC776.08c	Nrap (predicted)	protein_coding	II	-1
7	SPBP22H7.02c	RNA-binding protein Mrd1 (predicted)	protein_coding	II	-1

Table S1: mRNAs from Group A (cluster 7 in Table S4)

Let's look closer at the genes in Group B (which corresponds to cluster 17 in Table S4)

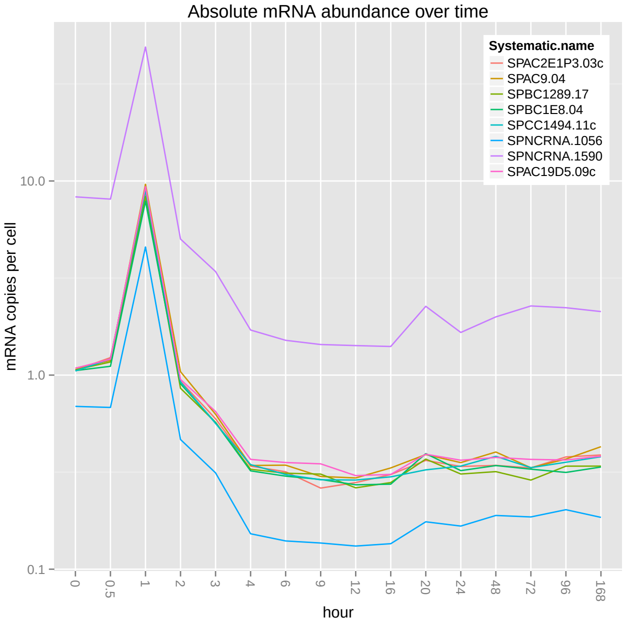

Figure S38: Absolute abundances of the 8 mRNAs in Group B. Note that the two non-coding RNAs (1056 and 1590) are at the lower and upper extremes.

c	Systematic ID	Description	Feature type	Chr	Str
17	SPAC19D5.09c	retrotransposable element/transposon Tf2-type	protein_coding	I	-1
17	SPAC2E1P3.03c	retrotransposable element/transposon Tf2-type	protein_coding	I	-1
17	SPAC9.04	retrotransposable element/transposon Tf2-type	protein_coding	I	1
17	SPBC1289.17	retrotransposable element/transposon Tf2-type	protein_coding	II	1
17	SPBC1E8.04	retrotransposable element/transposon Tf2-type	protein_coding	II	1
17	SPCC1494.11c	retrotransposable element/transposon Tf2-type	protein_coding	III	-1
17	SPNCRNA.1056	antisense RNA (predicted)	ncRNA	I	1
17	SPNCRNA.1590	antisense RNA (predicted)	ncRNA	II	-1

Table S2: mRNAs from Group B (cluster 17 in Table S4)

Noticing that six of the genes in this cluster 17 corresponded to retrotransposible elements, we downloaded the sequences of the genes in this cluster and aligned them using ClustalW2 (`http://www.ebi.ac.uk/Tools/msa/clustalw2/`) to get the following sequence identity matrix:

	SPBC1289.17	SPAC19D5.09c	SPBC1E8.04	SPAC9.04	SPCC1494.11c	SPAC2E1P3.03c	SPNCRNA.1590	SPNCRNA.1056
SPBC1289.17	100.0	99.8	99.5	99.6	99.6	99.6	75.6	55.6
SPAC19D5.09c	99.8	100.0	99.7	99.8	99.8	99.8	75.6	55.5
SPBC1E8.04	99.5	99.7	100.0	99.9	99.9	99.9	75.5	55.3
SPAC9.04	99.6	99.8	99.9	100.0	100.0	99.9	75.5	55.3
SPCC1494.11c	99.6	99.8	99.9	100.0	100.0	100.0	75.5	55.3
SPAC2E1P3.03c	99.6	99.8	99.9	99.9	100.0	100.0	75.6	55.4
SPNCRNA.1590	75.6	75.6	75.5	75.5	75.5	75.6	100.0	67.8
SPNCRNA.1056	55.6	55.5	55.3	55.3	55.3	55.4	67.8	100.0

Table S3: Percentage sequence identity for mRNAs from Group B (cluster 17 in Table S4)

This suggests that the highly proportional expression levels of the retrotransposible elements in this group are due to cross-hybridization on the microarray. In a sense, this finding provides a degree of biological validation for proportionality as a measure of association because it was made without advance knowledge of the high sequence similarity between the mRNAs in cluster 17.

So far we have looked closely at three clusters of mRNAs from Table S4: cluster 3 (96 mRNAs), cluster 7 (8 mRNAs) and cluster 17 (8 mRNAs). Because proportionality is a stricter relationship than correlation, we would not have found these particular relationships by looking at correlation of absolute values. To illustrate this fact, we can pick a gene g from one of those clusters and plot ϕ against the correlation coefficient ρ for all 3030 pairs (g, i) of mRNAs.

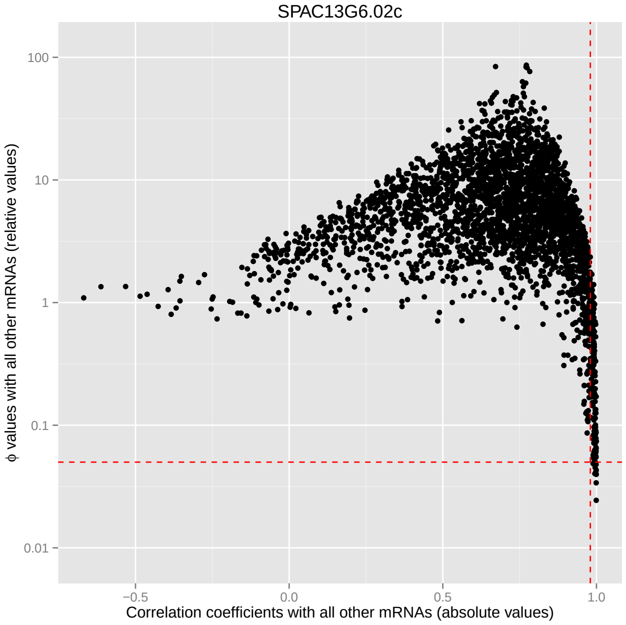

Figure S39: (ρ, ϕ) values for gene `SPAC13G6.02c` from the 96-gene cluster 3 in Table S4. The horizontal dashed line shows the value of $\phi = 0.05$ beneath which we deemed mRNA pairs to be strongly proportional. For comparison, the vertical dashed line is at a correlation of 0.98.

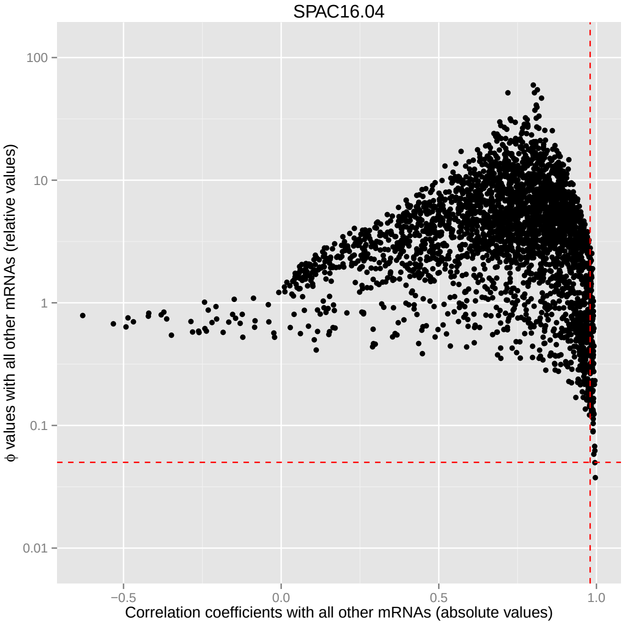

Figure S40: (ρ, ϕ) values for gene `SPBC1289.17` from the 8-gene cluster 7 in Table S4. The horizontal dashed line shows the value of $\phi = 0.05$ beneath which we deemed mRNA pairs to be strongly proportional. For comparison, the vertical dashed line is at a correlation of 0.98.

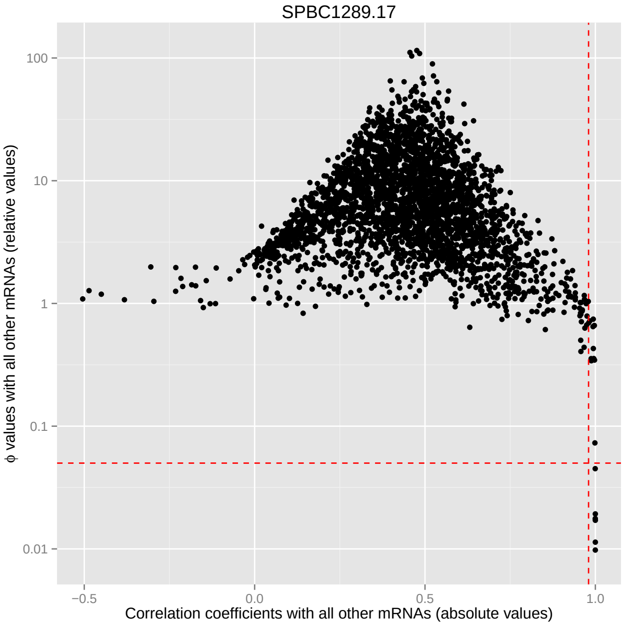

Figure S41: (ρ, ϕ) values for gene `SPBC1289.17` from the 8-gene cluster 17 in Table S4. The horizontal dashed line shows the value of $\phi = 0.05$ beneath which we deemed mRNA pairs to be strongly proportional. For comparison, the vertical dashed line is at a correlation of 0.98.

There are many more ways for variables to be correlated than there are for them to be proportional as we can see from the numbers of points to the right of the correlation cutoff in comparison to the number below

the proportionality cutoff in the preceding plots. When faced with a large multivariate dataset, looking first at the pairs of variables showing strong proportionality may be a more manageable analysis strategy than trying to make sense of all strongly correlated pairs from the get go.

S6 On the mathematics of different representations

S6.1 On the need to transform absolute data

This paper talks a lot about *absolute* data but glosses over precise details of this term. Absolute refers to measurements made on what Stevens [31] calls a *ratio scale*,

> ... [whose] numerical values can be transformed (as from inches to feet) only by multiplying each value by a constant. An absolute zero is always implied, even though the zero value on some scales (e.g. Absolute Temperature) may never be produced. All types of statistical measures are applicable to ratio scales, and only with these scales may we properly indulge in logarithmic transformations such as are involved in the use of decibels.

Absolute measurements take on *non-negative* values, i.e., zero or greater. In this paper, we avoid issues posed by zero values by requiring that the data be *positive*, i.e., greater than zero. Our interest centres on sets of absolute measurements (e.g., the set of yeast gene expression levels at a certain point in time) which means that we are working in the D-dimensional space of positive real numbers, written as $\mathbb{R}_{+}^{D}$ and also known as the *positive orthant*. The space of absolute data is a subset of $\mathbb{R}^{D}$, the Euclidean vector space of D-dimensions.

Compositional data analysis is founded on the idea of tranforming data (in this case relative abundances) from a restricted space (the simplex $\mathcal{S}^{D}$) into unrestricted Euclidean space so that all manner of statistical analyses can be performed without violating any of their assumptions, and secure in the knowledge that their results can be tranformed back into the simplex. To stay true to this principle with *absolute* data which also come from a restricted space (the positive orthant $\mathbb{R}_{+}^{D}$), we must also transform absolute data into unrestricted Euclidean space. Conventionally, this is done by taking logarithms of each measurement, but other approaches are valid—an issue which we discuss at length in [32].

There are some important open questions in this area, including

- How big a problem is it to apply a method intended for data in $\mathbb{R}^{D}$ to data that are constrained to lie in $\mathbb{R}_{+}^{D}$?
- Is it valid to draw inferences *on a limited range of data* in $\mathbb{R}_{+}^{D}$ using a method intended for data in $\mathbb{R}^{D}$?

In practical terms, this second point addresses the question of whether it is OK to use correlation on untransformed absolute abundances as we have done throughout the paper without making a fuss. We have done this (a) because we do not want to dilute the main point of the paper (to present and illustrate principles for analysing relative abundances) and (b) because this question is a research topic in its own right. Please take a look at Section S2.4 for further discussion of issues around interpretation, model fitting, and the nature of error in the system under study when working with log-transformed data.

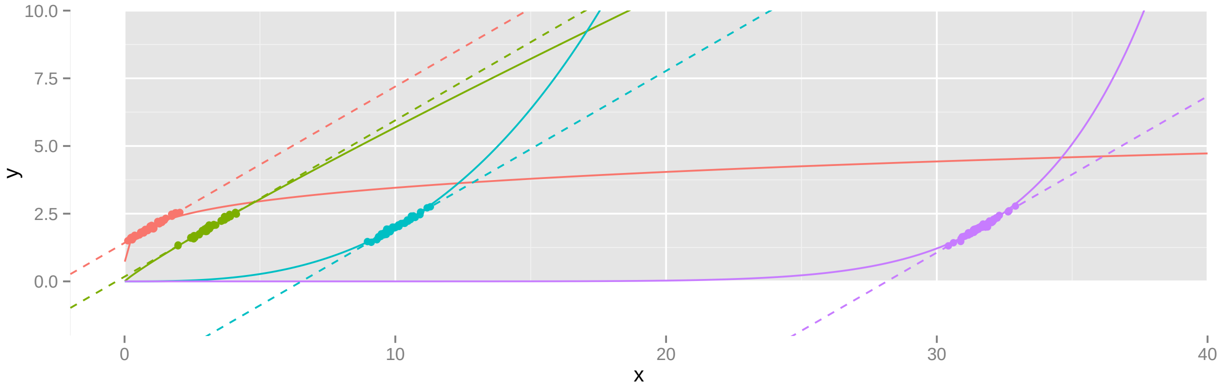

Figure S42: Four sets of points whose mean x values are $10^0, 10^{0.5}, 10^1, 10^{1.5}$, respectively; whose mean y values are 1; and that fit lines of slope 30°. The dashed lines show linear models fitted to the points; the solid lines show the fits of log-linear models.

The late George Box wrote "Remember that all models are wrong; the practical question is how wrong do they have to be to not be useful." With that in mind, we give some examples to illustrate "how wrong" it can be to use methods intended for data in $\mathbb{R}^D$ on data constrained to lie in $\mathbb{R}^D_+$. To do this, we use the four sets of points shown in Figure S42. These sets of points were chosen to fit lines with the same slope but different intercepts. Importantly, each set of points covers a limited range of x and y.

If we knew that these points lay on an *interval scale* [31, Table 1] (i.e., the process that generated them could, in theory, generate points anywhere in $\mathbb{R}^2$) then we would be justified to model them using linear relationships as shown by the dashed lines in Figure S42. You can see these linear models imply that data could take negative values if we extrapolated beyond the ranges we had observed.

However, if we knew that these points lay on a *ratio scale*, data would be constrained to the positive orthant $\mathbb{R}^2_+$ (which we have indicated with the grey background). This constraint warrants that the data be logarithmically transformed before applying statistical methods (including correlation) that assume the data can lie anywhere in Euclidean space. The solid lines in Figure S42 show the models obtained when we first logarithmically transform the data, then fit linear models to the transformed values, and finally transform everything back into the original sample space by taking antilogarithms. Now, if we use these models to extrapolate beyond the ranges of the observed data, we see that the predicted values remain consistent with the ratio scale, i.e., they remain in $\mathbb{R}^2_+$. The beauty of this approach is that is it safe for all possible values in the sample space.

Now, let us consider the merits of these approaches within the ranges of the data that were actually observed.

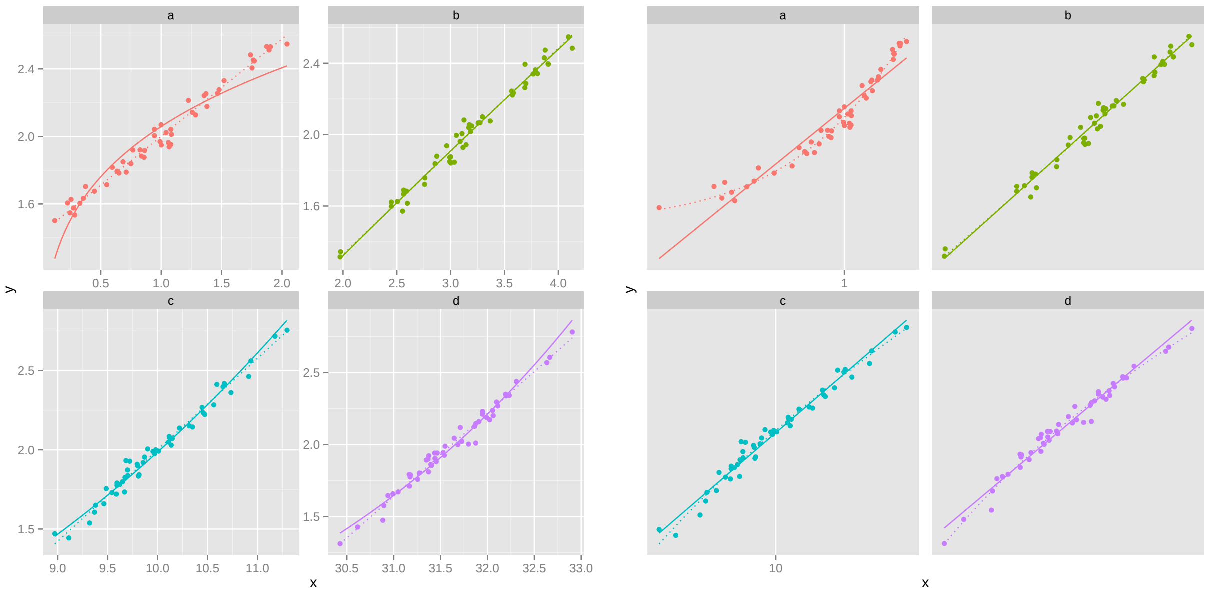

Figure S43: The four plots on the left show the sets of points from Figure S42 on the original linear scale; the four plots on the right show the points on a log-log scale. This figure presents a *local* view of the data in Figure S42—it concentrates our attention on the ranges of values that were actually observed—whereas Figure S42 gives a *global* view, showing how the data and their models sit within 2D Euclidean space.

If we concentrate our attention on the ranges of values that were actually observed, we see situations where the linear and the log-linear models are quite different (Group a in Figure S43), almost identical (Group b), and somewhere in between (Groups c and d).

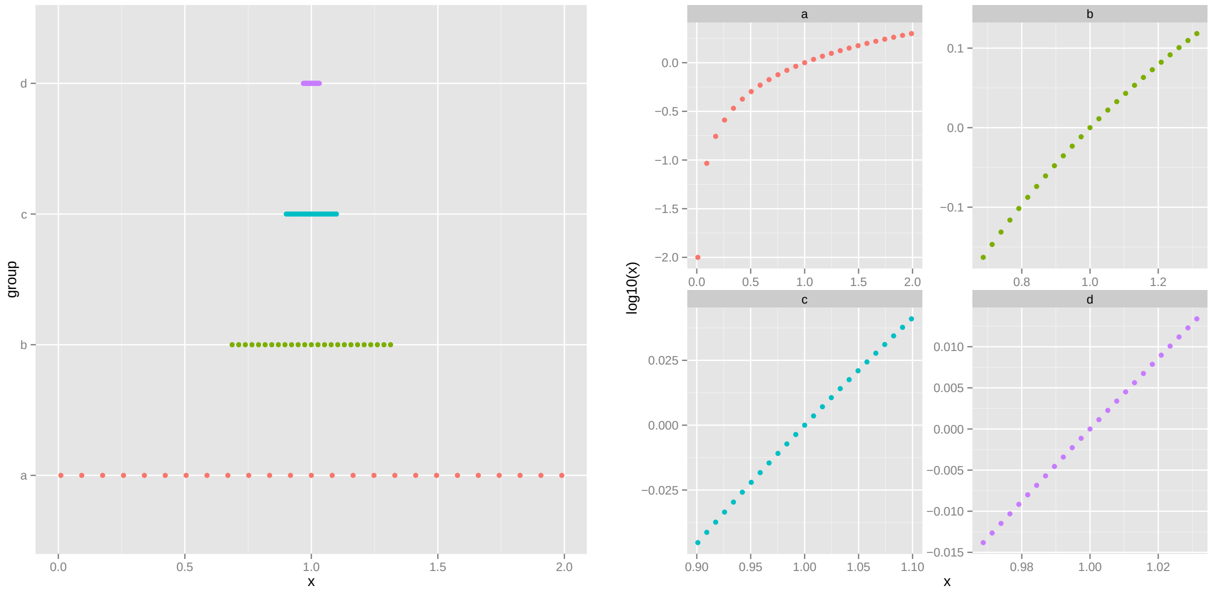

Figure S44: The left plot shows four sets of points with different dispersion. The right plots show how the more dispersed data are, the less linear the results of logarithmic tranformation. Conversely, the less dispersed data are, the more linearly the logarithmic tranformation behaves.

The adequacy of fit depends on the univariate dispersion of the data: the more dispersed data are, the less linear the results of logarithmic tranformation (Figure S44)). Note also that if variables x and y behave proportionally (as is almost the case with the green points in Figures S42 and S43), both the linear and log-linear models will fit equally well. The coefficient of variation is an appropriate measure of dispersion:

```
##   group coeff.of.variation
## 1     a            0.60718
## 2     b            0.19201
## 3     c            0.06072
## 4     d            0.01920
```

In summary, modeling involves choices, but it is important that these choices are well-informed. When working with absolute amounts (i.e., values that exist on a ratio scale and which, by definition, are constrained to be greater than or equal to zero), one should log-transform the data prior to analysis with methods that assume the data exist in $\mathbb{R}^D$. However, if the ratio scale data show little dispersion and there is no intent to extrapolate beyond the range of the observed data, one can apply $\mathbb{R}^D$ analysis methods on the understanding that their results are only valid *locally* (i.e., over the range of the observed data). To be on the safe side, applying and investigating logarithmic transformation is always advisable, because nobody knows how small is “small”.

S6.2 On the need to use the clr representation of data

In introducing the concept of our goodness-of-fit-to-proportionality statistic we have used $\phi(\log x, \log y)$ to emphasise its relationship to Aitchison's logratio variance $\text{var}(\log(x/y))$. However, when it came to actually looking for proportionality between the relative abundances of different yeast mRNAs, we first applied the *centered logratio* (clr) transformation to the data (see Section S5.4). Why?

clr transformation maps D-component compositional data from the simplex $\mathcal{S}^D$ to its representation in a plane in $\mathbb{R}^D$ (i.e., a $D-1$ dimensional subspace). With this representation, we can analyse the data using familiar methods for Euclidean spaces and, if necessary, transform results back to the simplex from which the original compositions came [28].

The clr representation of compositional data is important in testing the hypothesis of proportionality between two components (Section S5.4) because it ensures that the residuals are correctly scaled. However, if we are interested in goodness of fit to proportionality rather than hypothesis testing, why not just work with the logs of the components, just as we do in the variation array?

To answer that, we need to introduce some more precise notation. The data of interest is $\mathbf{X}$, an $N \times D$ matrix of N observations of D components in which the ith observation is the composition $\mathbf{x}_i = [x_{i1}, x_{i2}, \ldots, x_{iD}]$. We use the dot ($\bullet$) to indicate which dimension a mean or variance is being calculated on, so if we write

$$\text{clr}(\mathbf{X}) = \begin{bmatrix} \text{clr}(\mathbf{x}_1) \\ \text{clr}(\mathbf{x}_2) \\ \vdots \\ \text{clr}(\mathbf{x}_N) \end{bmatrix}$$

then $\text{clr}(x_{\bullet j})$ is the jth column of $\text{clr}(\mathbf{X})$. Here, we will use both j and k to index different columns (components).

Now we can ask the question more precisely: why not use $\phi(\log x_{\bullet j}, \log x_{\bullet k})$ instead of $\phi(\text{clr}(x_{\bullet j}), \text{clr}(x_{\bullet k}))$? To answer that, we need to understand the relationship between the value of these two different expressions. We have already shown that we can factorise logratio variance as

$$\text{var}\left(\log \frac{x_{\bullet j}}{x_{\bullet k}}\right) = \text{var}(\log x_{\bullet j}) \cdot \phi(\log x_{\bullet j}, \log x_{\bullet k})$$

but we can also write

$$\begin{aligned} \text{var}\left(\log \frac{x_{\bullet j}}{x_{\bullet k}}\right) &= \text{var}\left(\log\left(\frac{x_{\bullet j}}{\text{g}_\text{m}(\mathbf{x}_\bullet)} \cdot \frac{\text{g}_\text{m}(\mathbf{x}_\bullet)}{x_{\bullet k}}\right)\right) \\ &= \text{var}\left(\log\left(\frac{x_{\bullet j}}{\text{g}_\text{m}(\mathbf{x}_\bullet)}\right)\right) \cdot \phi(\text{clr}(x_{\bullet j}), \text{clr}(x_{\bullet k})) \\ &= \text{var}(\log x_{\bullet j}) \cdot \phi(\log x_{\bullet j}, \log \text{g}_\text{m}(\mathbf{x}_\bullet)) \cdot \phi(\text{clr}(x_{\bullet j}), \text{clr}(x_{\bullet k})) \end{aligned}$$

so that we can see

$$\phi(\log x_{\bullet j}, \log x_{\bullet k}) = \phi(\log x_{\bullet j}, \log \text{g}_\text{m}(\mathbf{x}_\bullet)) \cdot \phi(\text{clr}(x_{\bullet j}), \text{clr}(x_{\bullet k})). \tag{2}$$

So the problem with using $\phi(\log x_{\bullet j}, \log x_{\bullet k})$ as a measure of the proportionality between components j and k is that it contains a positive scaling factor $\phi(\log x_{\bullet j}, \log \text{g}_\text{m}(\mathbf{x}_\bullet))$ that is particular to component j. Thus, if we were to look at the proportionality between two different components, say m and n, the value $\phi(\log x_{\bullet m}, \log x_{\bullet n})$ would be on a different scale and not directly comparable to $\phi(\log x_{\bullet j}, \log x_{\bullet k})$.

Let's look at this in practice. For convenience, we use a subset of the yeast data, including some mRNAs that are known to be behaving proportionally:

```
X            <- Rel.t[,c(1,2, grep("SPNCRNA.1056|SPNCRNA.1590", names(Rel.t)))]
```

Next we calculate $\phi(\log x_{\bullet j}, \log x_{\bullet k})$

```
X.vlr       <- variation(acomp(X))

X.log       <- log(X)
X.log.var   <- apply(X.log, 2, var)
X.log.phi   <- sweep(X.vlr, 2, X.log.var, FUN="/")
```

then $\phi(\mathrm{clr}(x_{\bullet j}), \mathrm{clr}(x_{\bullet k}))$

```
X.clr       <- clr(X)
X.clr.var   <- apply(X.clr, 2, var)
X.clr.phi   <- sweep(X.vlr, 2, X.clr.var, FUN="/")
```

If we look at the ratio of these two $\phi()$ matrices, we can see that the columns are scaled by different factors

```
X.log.phi/X.clr.phi

##                SPAC1002.02 SPAC1002.03c SPNCRNA.1056 SPNCRNA.1590
## SPAC1002.02            NaN        7.611       0.2951       0.2889
## SPAC1002.03c         1.186          NaN       0.2951       0.2889
## SPNCRNA.1056         1.186        7.611          NaN       0.2889
## SPNCRNA.1590         1.186        7.611       0.2951          NaN
```

and these scaling factors relate to $\phi(\log x_{\bullet j}, \log \mathrm{g_m}(\mathbf{x}_{\bullet}))$:

```
Xgm         <- cbind(X, gm=geometricmeanRow(X))
Xgm.vlr     <- variation(acomp(Xgm))
Xgm.log     <- log(Xgm)
Xgm.log.var <- apply(Xgm.log, 2, var)
Xgm.log.phi <- sweep(Xgm.vlr, 2, Xgm.log.var, FUN="/")
Xgm.log.phi["gm", -5, drop=FALSE]

##    SPAC1002.02 SPAC1002.03c SPNCRNA.1056 SPNCRNA.1590
## gm       1.186        7.611       0.2951       0.2889
```

Graphically, the difference between clr and log-transformed components of $\mathbf{x}_i$ is a translation of $\log \mathrm{g_m}(\mathbf{x}_i)$. For pairs of variables, this amounts to a shift along lines of slope 1 as shown here:

```
X.12.df  <- rbind(
  data.frame(X.log[,1:2], transformation="log", timepoint=rownames(X.log)),
  data.frame(X.clr[,1:2], transformation="clr", timepoint=rownames(X.clr))
)

ggplot(data=X.12.df, aes(x=SPAC1002.02, y=SPAC1002.03c, group=timepoint)) +
  geom_line(aes(group=timepoint), colour="grey") +
  geom_point(aes(colour=transformation), size=3) +
  geom_point(data=subset(X.12.df, timepoint=="timepoint1"),  shape=0, size=4) +
  coord_equal()
```

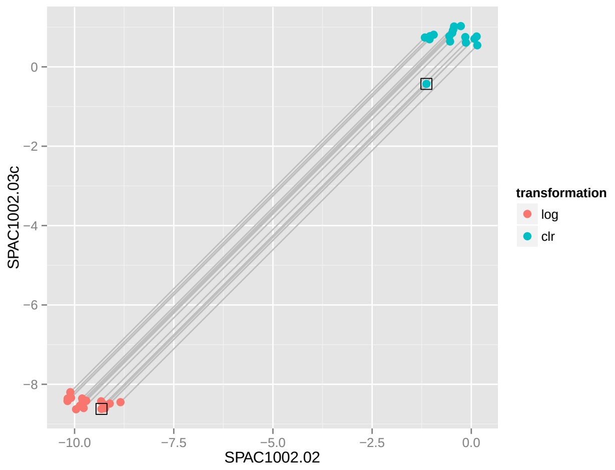

Figure S45: The clr and log-transformed values of `SPAC1002.02` and `SPAC1002.03c` relative abundances. The grey lines connect the values at each timepoint. Values at timepoint 1 are boxed.

This plot highlights that $\log \mathrm{g_m}(\mathbf{x}_i)$ is different for each observation: the boxed points show that the geometric mean of the composition at timepoint 1 is a lot different from the other timepoints.

The other thing to note is that points that are spread out along the line of slope 1 after log transformation will still be spread out along that same line after clr transformation:

```
X.34.df  <- rbind(
  data.frame(X.log[,3:4], transformation="log", timepoint=rownames(X.log)),
  data.frame(X.clr[,3:4], transformation="clr", timepoint=rownames(X.clr))
)

ggplot(data=X.34.df, aes(x=SPNCRNA.1056, y=SPNCRNA.1590, group=timepoint)) +
  geom_line(aes(group=timepoint), colour="grey") +
  geom_point(aes(colour=transformation), size=3) +
  coord_equal()
```

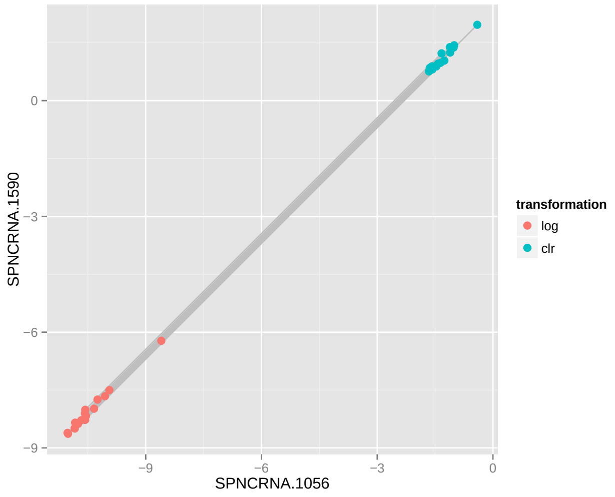

Figure S46: The clr and log-transformed values of `SPNCRNA.1056` and `SPNCRNA.1590` relative abundances. The grey lines connect the values at each timepoint.

This shows graphically that when $\phi(\text{clr}(x_{\bullet j}), \text{clr}(x_{\bullet k}))$ is close to zero, so is $\phi(\log x_{\bullet j}, \log x_{\bullet k})$.

We have used goodness of fit to proportionality rather than hypothesis testing because we are interested in finding pairs of components whose behaviour is strongly proportional, not whether the data is consistent with the hypothesis of unit slope. While we can give a clear explanation as to the relationship between $\phi(\mathrm{clr}(x_{\bullet j}), \mathrm{clr}(x_{\bullet k}))$ and $\phi(\log x_{\bullet j}, \log x_{\bullet k})$, we are not able to do so for the p-values that arise from the hypothesis test of unit slope on the log and -clr transformed data. All we can say at this stage is that they are different:

```
X.log.sma   <- sma.df(X.log)
X.clr.sma   <- sma.df(X.clr)
round(X.log.sma$p,3)

##              SPAC1002.02 SPAC1002.03c SPNCRNA.1056 SPNCRNA.1590
## SPAC1002.02        1.000            0        0.216        0.210
## SPAC1002.03c       0.000            1        0.000        0.000
## SPNCRNA.1056       0.216            0        1.000        0.872
## SPNCRNA.1590       0.210            0        0.872        1.000

round(X.clr.sma$p,3)

##              SPAC1002.02 SPAC1002.03c SPNCRNA.1056 SPNCRNA.1590
## SPAC1002.02        1.000        0.214        0.015        0.018
## SPAC1002.03c       0.214        1.000        0.881        0.854
## SPNCRNA.1056       0.015        0.881        1.000        0.923
## SPNCRNA.1590       0.018        0.854        0.923        1.000
```

S7 Pombase information on mRNAs behaving proportionally

c	Systematic ID	Description	Feature type	Chr	Str
1	SPAC1071.07c	40S ribosomal protein S15 (predicted)	protein_coding	I	-1
1	SPAC22H12.04c	40S ribosomal protein S3a (predicted)	protein_coding	I	-1
1	SPAC2C4.16c	40S ribosomal protein S8 (predicted)	protein_coding	I	-1
1	SPBC19F8.08	40S ribosomal protein S4 (predicted)	protein_coding	II	1
1	SPBC25H2.05	nascent polypeptide-associated complex alpha subunit Egd2	protein_coding	II	-1
2	SPAC1071.11	NADH-dependent flavin oxidoreductase (predicted)	protein_coding	I	1
2	SPAC23A1.05	serine palmitoyltransferase subunit A (predicted)	protein_coding	I	1
2	SPBC21C3.15c	aldehyde dehydrogenase (predicted)	protein_coding	II	-1
2	SPBC543.08	phosphoinositide biosynthesis protein (predicted)	protein_coding	II	1
3	SPAC10F6.10	protein kinase, RIO family (predicted)	protein_coding	I	1
3	SPAC13G6.02c	40S ribosomal protein S3a	protein_coding	I	-1
3	SPAC13G6.07c	40S ribosomal protein S6	protein_coding	I	-1
3	SPAC144.11	40S ribosomal protein S11 (predicted)	protein_coding	I	1
3	SPAC1486.09	ribosome biogenesis protein Nob1 (predicted)	protein_coding	I	1
3	SPAC1565.05	sequence orphan	protein_coding	I	1
3	SPAC15A10.04c	EF-1 alpha binding zinc finger protein Zpr1 (predicted)	protein_coding	I	-1
3	SPAC1687.06c	60S ribosomal protein L28/L44 (predicted)	protein_coding	I	-1
3	SPAC1783.08c	60S ribosomal protein L15b (predicted)	protein_coding	I	-1
3	SPAC17H9.05	rRNA processing protein Ebp2 (predicted)	protein_coding	I	1
3	SPAC1805.13	60S ribosomal protein L14 (predicted)	protein_coding	I	1
3	SPAC18B11.06	U3 snoRNP-associated protein Lcp5 (predicted)	protein_coding	I	-1
3	SPAC18G6.14c	40S ribosomal protein S7 (predicted)	protein_coding	I	-1
3	SPAC19B12.04	40S ribosomal protein S30 (predicted)	protein_coding	I	1
3	SPAC1B9.03c	RNA-binding protein involved in ribosomal large subunit assembly and maintenance (predicted)	protein_coding	I	1
3	SPAC1F7.13c	60S ribosomal protein L8 (predicted)	protein_coding	I	-1
3	SPAC20G8.09c	ribosome biogenesis ATPase	protein_coding	I	-1
3	SPAC222.06	nuclear HMG-like acidic protein Mak16 (predicted)	protein_coding	I	1
3	SPAC23A1.03	adenine phosphoribosyltransferase (APRT) (predicted)	protein_coding	I	1
3	SPAC23A1.08c	60S ribosomal protein L34	protein_coding	I	-1
3	SPAC23A1.11	60S ribosomal protein L13/L16 (predicted)	protein_coding	I	1
3	SPAC24H6.07	40S ribosomal protein S9	protein_coding	I	-1
3	SPAC26A3.07c	60S ribosomal protein L11 (predicted)	protein_coding	I	-1
3	SPAC31G5.03	40S ribosomal protein S11 (predicted)	protein_coding	I	1
3	SPAC328.10c	40S ribosomal protein S5 (predicted)	protein_coding	I	-1
3	SPAC3A12.10	60S ribosomal protein L20a (predicted)	protein_coding	I	1
3	SPAC3F10.16c	GTP binding protein, HSR1-related (predicted)	protein_coding	I	-1
3	SPAC3G9.03	60S ribosomal protein L23	protein_coding	I	-1
3	SPAC3H5.10	60S ribosomal protein L32 (predicted)	protein_coding	I	-1
3	SPAC3H5.12c	60S ribosomal protein L5 (predicted)	protein_coding	I	1
3	SPAC4F10.06	ribosome small subunit biogenesis protein, BUD22 family (predicted)	protein_coding	I	1
3	SPAC4G9.16c	60S ribosomal protein L9	protein_coding	I	-1
3	SPAC521.05	40S ribosomal protein S8 (predicted)	protein_coding	I	1
3	SPAC5D6.01	40S ribosomal protein S15a (predicted)	protein_coding	I	-1
3	SPAC644.15	60S acidic ribosomal protein A1	protein_coding	I	1
3	SPAC664.05	60S ribosomal protein L13 (predicted)	protein_coding	I	1
3	SPAC6B12.09	tRNA m(1)G methyltransferase Trm10	protein_coding	I	1
3	SPAC6F6.03c	ribosome export GTPase (predicted)	protein_coding	I	-1
3	SPAC6G9.09c	60S ribosomal protein L24 (predicted)	protein_coding	I	-1
3	SPAC8C9.08	40S ribosomal protein S5 (predicted)	protein_coding	I	1
3	SPAC959.07	40S ribosomal protein S4 (predicted)	protein_coding	I	1
3	SPAC9G1.03c	60S ribosomal protein L30 (predicted)	protein_coding	I	-1
3	SPAPB17E12.05	60S ribosomal protein L37 (predicted)	protein_coding	I	1
3	SPBC11G11.05	DNA-directed RNA polymerase I complex subunit Rpa34 (predicted)	protein_coding	II	1
3	SPBC1539.10	ribosome biogenesis protein Nop16 (predicted)	protein_coding	II	1
3	SPBC16D10.11c	40S ribosomal protein S18 (predicted)	protein_coding	II	-1
3	SPBC16G5.14c	40S ribosomal protein S3 (predicted)	protein_coding	II	-1
3	SPBC1711.06	60S ribosomal protein L4 (predicted)	protein_coding	II	1
3	SPBC1711.16	WD repeat protein (predicted)	protein_coding	II	1
3	SPBC17G9.07	40S ribosomal protein S24 (predicted)	protein_coding	II	1
3	SPBC17G9.10	60S ribosomal protein L11 (predicted)	protein_coding	II	1
3	SPBC18E5.04	60S ribosomal protein L10	protein_coding	II	1

c	Systematic ID	Description	Feature type	Chr	Str
3	SPBC18H10.13	40S ribosomal protein S14 (predicted)	protein_coding	II	1
3	SPBC21B10.10	40S ribosomal protein S4 (predicted)	protein_coding	II	-1
3	SPBC21C3.13	40S ribosomal protein S19 (predicted)	protein_coding	II	1
3	SPBC26H8.08c	GTPase Grn1	protein_coding	II	-1
3	SPBC29A3.12	40S ribosomal protein S9 (predicted)	protein_coding	II	1
3	SPBC29B5.03c	60S ribosomal protein L26 (predicted)	protein_coding	II	-1
3	SPBC2F12.07c	60S ribosomal protein L8 (predicted)	protein_coding	II	1
3	SPBC31E1.06	GTP binding protein Bms1 (predicted)	protein_coding	II	1
3	SPBC336.10c	translation elongation factor eIF5A (predicted)	protein_coding	II	-1
3	SPBC365.03c	60S ribosomal protein L21 (predicted)	protein_coding	II	-1
3	SPBC405.07	60S ribosomal protein L36	protein_coding	II	1
3	SPBC409.15	rRNA processing protein Tsr2 (predicted)	protein_coding	II	1
3	SPBC4F6.07c	ATP-dependent RNA helicase Mak5 (predicted)	protein_coding	II	-1
3	SPBC649.02	40S ribosomal protein S19 (predicted)	protein_coding	II	1
3	SPBC685.07c	60S ribosomal protein L27	protein_coding	II	-1
3	SPBC776.01	60S ribosomal protein L29	protein_coding	II	1
3	SPBC776.11	60S ribosomal protein L27/L28	protein_coding	II	1
3	SPBC800.04c	60S ribosomal protein L37a (predicted)	protein_coding	II	-1
3	SPBC839.04	60S ribosomal protein L8 (predicted)	protein_coding	II	1
3	SPBC839.05c	40S ribosomal protein S17 (predicted)	protein_coding	II	-1
3	SPBC839.13c	60S ribosomal protein L13/L16 (predicted)	protein_coding	II	-1
3	SPBC947.07	ribosome biogenesis protein Rrp14 (predicted)	protein_coding	II	-1
3	SPBP4H10.13	40S ribosomal protein S23 (predicted)	protein_coding	II	1
3	SPBP8B7.16c	ATP-dependent RNA helicase Dbp2	protein_coding	II	-1
3	SPBP8B7.20c	RNA methyltransferase Nop2 (predicted)	protein_coding	II	-1
3	SPCC1183.08c	60S ribosomal protein L10a	protein_coding	III	-1
3	SPCC1223.05c	60S ribosomal protein L37 (predicted)	protein_coding	III	-1
3	SPCC1259.01c	40S ribosomal protein S18 (predicted)	protein_coding	III	-1
3	SPCC14G10.02	ribosome biogenesis protein Urb1 (predicted)	protein_coding	III	1
3	SPCC1682.14	60S ribosomal protein L19	protein_coding	III	1
3	SPCC16C4.13c	60S ribosomal protein L12.1/L12A	protein_coding	III	-1
3	SPCC24B10.09	40S ribosomal protein S17 (predicted)	protein_coding	III	1
3	SPCC330.09	rRNA processing protein Enp2 (predicted)	protein_coding	III	1
3	SPCC364.03	60S ribosomal protein L17 (predicted)	protein_coding	III	-1
3	SPCC576.08c	40S ribosomal protein S2 (predicted)	protein_coding	III	-1
3	SPCC576.09	40S ribosomal protein S20 (predicted)	protein_coding	III	1
3	SPCC576.11	60S ribosomal protein L15 (predicted)	protein_coding	III	1
3	SPCC613.06	60S ribosomal protein L9	protein_coding	III	1
3	SPCC613.08	CDK regulator, involved in ribosome export (predicted)	protein_coding	III	1
3	SPCC622.18	60S ribosomal protein L6 (predicted)	protein_coding	III	1
3	SPCC962.04	40S ribosomal protein S12 (predicted)	protein_coding	III	1
3	SPCP1E11.09c	60S acidic ribosomal protein Rpp1-3	protein_coding	III	-1
3	SPCP31B10.08c	60S ribosomal protein L35a	protein_coding	III	-1
3	SPCPB16A4.04c	tRNA (guanine-N7-)-methyltransferase catalytic subunit Trm8 (predicted)	protein_coding	III	-1
4	SPAC1399.01c	membrane transporter (predicted)	protein_coding	I	1
4	SPAC1F7.12	aldose reductase ARK13 family YakC	protein_coding	I	1
5	SPAC14C4.09	glucan endo-1,3-alpha-glucosidase Agn1	protein_coding	I	1
5	SPAPYUG7.03c	medial ring protein Mid2	protein_coding	I	-1
5	SPBC3E7.12c	chitin synthase regulatory factor Cfh4 (predicted)	protein_coding	II	-1
5	SPCC18.01c	beta-glucosidase Adg3 (predicted)	protein_coding	III	-1
6	SPAC15E1.06	retromer complex subunit Vps29	protein_coding	I	1
6	SPAC1F5.10	ATP-dependent RNA helicase (predicted)	protein_coding	I	-1
7	SPAC16.04	tRNA dihydrouridine synthase Dus3 (predicted)	protein_coding	I	1
7	SPAC1687.11	rRNA methyltransferase Spb1 (predicted)	protein_coding	I	1
7	SPAC16C9.03	export adaptor Nmd3 (predicted)	protein_coding	I	1
7	SPAC19A8.07c	U3 snoRNP-associated protein Imp4 (predicted)	protein_coding	I	1
7	SPBC106.14c	SDA1 family protein (predicted)	protein_coding	II	-1
7	SPBC1105.01	rRNA processing protein Rrp12-like (predicted)	protein_coding	II	1
7	SPBC776.08c	Nrap (predicted)	protein_coding	II	-1
7	SPBP22H7.02c	RNA-binding protein Mrd1 (predicted)	protein_coding	II	-1
8	SPAC16C9.05	Clr6 histone deacetylase associated PHD protein-1 Cph1	protein_coding	I	1
8	SPAC4C5.02c	GTPase Ryh1	protein_coding	I	-1
9	SPAC16E8.03	glucosamine-phosphate N-acetyltransferase (predicted)	protein_coding	I	1
9	SPBC337.12	human ZC3H3 homolog	protein_coding	II	1
10	SPAC1782.06c	prohibitin Phb1 (predicted)	protein_coding	I	-1
10	SPAC6G9.08	ubiquitin C-terminal hydrolase Ubp6	protein_coding	I	1

c	Systematic ID	Description	Feature type	Chr	Str
11	SPAC17A2.03c	V-type ATPase V0 subunit d (predicted)	protein_coding	I	-1
11	SPBC16H5.12c	conserved fungal protein	protein_coding	II	1
11	SPCC613.10	ubiquinol-cytochrome-c reductase complex core protein Qcr2 (predicted)	protein_coding	III	1
12	SPAC1805.08	dynein light chain Dlc1	protein_coding	I	1
12	SPBC21C3.04c	mitochondrial ribosomal protein subunit L34 (predicted)	protein_coding	II	-1
13	SPAC1834.01	translation release factor eRF1	protein_coding	I	1
13	SPCC1259.03	DNA-directed RNA polymerase complex I subunit Rpa12	protein_coding	III	1
14	SPAC186.03	L-asparaginase (predicted)	protein_coding	I	1
14	SPBPB21E7.09	L-asparaginase (predicted)	protein_coding	II	1
15	SPAC18B11.09c	serine O-acetyltransferase activity (predicted)	protein_coding	I	1
15	SPNCRNA.1291	intergenic RNA (predicted)	ncRNA	III	1
15	SPNCRNA.1573	antisense RNA (predicted)	ncRNA	II	-1
15	SPNCRNA.519	intergenic RNA (predicted)	ncRNA	III	1
16	SPAC19D5.05c	U3 snoRNP-associated protein Imp3 (predicted)	protein_coding	I	-1
16	SPAC959.03c	U3 snoRNP-associated protein Utp7 (predicted)	protein_coding	I	-1
17	SPAC19D5.09c	retrotransposable element/transposon Tf2-type	protein_coding	I	-1
17	SPAC2E1P3.03c	retrotransposable element/transposon Tf2-type	protein_coding	I	-1
17	SPAC9.04	retrotransposable element/transposon Tf2-type	protein_coding	I	1
17	SPBC1289.17	retrotransposable element/transposon Tf2-type	protein_coding	II	1
17	SPBC1E8.04	retrotransposable element/transposon Tf2-type	protein_coding	II	1
17	SPCC1494.11c	retrotransposable element/transposon Tf2-type	protein_coding	III	-1
17	SPNCRNA.1056	antisense RNA (predicted)	ncRNA	I	1
17	SPNCRNA.1590	antisense RNA (predicted)	ncRNA	II	-1
18	SPAC19G12.16c	conserved fungal protein Adg2	protein_coding	I	-1
18	SPAC821.09	endo-1,3-beta-glucanase Eng1	protein_coding	I	1
19	SPAC1D4.08	CDP-diacylglycerol–inositol 3-phosphatidyltransferase Pis1 (predicted)	protein_coding	I	1
19	SPAC664.01c	HP1 family chromodomain protein Swi6	protein_coding	I	-1
20	SPAC1F12.02c	translationally controlled tumor protein homolog (predicted)	protein_coding	I	-1
20	SPCC1259.02c	Endoplasmic Reticulum metallopeptidase 1 (predicted)	protein_coding	III	-1
21	SPAC1F12.04c	conserved fungal protein	protein_coding	I	-1
21	SPBC32H8.12c	actin Act1	protein_coding	II	-1
21	SPBC9B6.08	clathrin light chain	protein_coding	II	1
21	SPBPJ4664.01	decaprenyl diphosphate synthase subunit Dps1	protein_coding	II	1
22	SPAC20G4.05c	UPF0061 family protein	protein_coding	I	-1
22	SPAC57A7.13	RNA-binding protein, involved in splicing (predicted)	protein_coding	I	-1
22	SPBC30D10.12c	mitochondrial ribosomal protein subunit S27 (predicted)	protein_coding	II	1
22	SPBC6B1.09c	Mre11 complex subunit Nbs1	protein_coding	II	-1
23	SPAC20G4.07c	C-24(28) sterol reductase Sts1	protein_coding	I	-1
23	SPAC9.02c	polyamine N-acetyltransferase (predicted)	protein_coding	I	-1
23	SPBC16A3.15c	tubulin alpha 1	protein_coding	II	1
24	SPAC212.09c	pseudogene	pseudogene	I	1
24	SPAC750.08c	NAD-dependent malic enzyme (predicted), partial	protein_coding	I	-1
24	SPBCPT2R1.07c	pseudogene malic enzyme (predicted)	pseudogene	II	-1
25	SPAC212.10	pseudogene malic acid transport protein	pseudogene	I	-1
25	SPBCPT2R1.10	pseudogene	pseudogene	II	1
26	SPAC30D11.10	DNA recombination protein Rad52 (previously Rad22)	protein_coding	I	-1
26	SPCC1620.07c	lunapark homolog Lnp1	protein_coding	III	-1
27	SPAC31G5.14	glycine decarboxylase T subunit (predicted)	protein_coding	I	1
27	SPAC4G9.10	ornithine carbamoyltransferase Arg3	protein_coding	I	1
28	SPAC4F8.14c	3-hydroxy-3-methylglutaryl-CoA synthase	protein_coding	I	1
28	SPBC543.02c	DNAJ/TPR domain protein DNAJC7 family	protein_coding	II	-1
29	SPAC513.01c	translation elongation factor 2 (EF-2) Eft2,A	protein_coding	I	-1
29	SPCP31B10.07	translation elongation factor 2 (EF-2) Eft2,B	protein_coding	III	1
30	SPAC5D6.04	auxin family transmembrane transporter (predicted)	protein_coding	I	-1
30	SPAC630.05	GTPase activating protein Gyp7 (predicted)	protein_coding	I	1
31	SPAC5H10.08c	pantoate-beta-alanine ligase	protein_coding	I	-1
31	SPBC2A9.05c	Golgi transport protein Tvp23 (predicted)	protein_coding	II	-1
32	SPAC637.03	conserved fungal protein	protein_coding	I	1
32	SPCC338.12	proteinase B inhibitor Pbi2 (predicted)	protein_coding	III	-1
33	SPAC637.06	alpha-1,2-galactosyltransferase (predicted)	protein_coding	I	1
33	SPBC31F10.16	ChAPs family protein (predicted)	protein_coding	II	1
33	SPCC1795.03	UDP-galactose transporter Gms1	protein_coding	III	-1
34	SPAC644.12	cell division control protein, splicing factor Cdc5	protein_coding	I	1

c	Systematic ID	Description	Feature type	Chr	Str
34	SPCC16C4.12	NatB N-acetyltransferase complex catalytic subunit Naa20 (predicted)	protein_coding	III	1
35	SPAC8F11.04	U3 snoRNP-associated protein Cic1/Utp30 family (predicted)	protein_coding	I	1
35	SPBC3B9.01	Hsp70 nucleotide exchange factor (predicted)	protein_coding	II	1
36	SPACUNK4.11c	nuclear exosome-associated RNA binding protein Mpp6	protein_coding	I	1
36	SPCC16C4.05	RNase P and RNase MRP subunit (predicted)	protein_coding	III	1
37	SPBC1718.06	mitochondrial dynamin family GTPase Msp1	protein_coding	II	1
37	SPCP31B10.04	conserved fungal protein	protein_coding	III	1
38	SPBC1773.10c	cytoplasmic asparagine-tRNA ligase Nrs1 (predicted)	protein_coding	II	-1
38	SPBC24C6.04	delta-1-pyrroline-5-carboxylate dehydrogenase (predicted)	protein_coding	II	1
39	SPBC19C2.13c	cytosolic thiouridylase subunit Ctu2	protein_coding	II	-1
39	SPBC3B8.09	U3 snoRNP-associated protein Utp3 (predicted)	protein_coding	II	-1
40	SPBC30D10.18c	60S ribosomal protein L10a	protein_coding	II	1
40	SPCC24B10.18	human Leydig cell tumor 10 kDa protein homolog	protein_coding	III	1
41	SPBC32H8.05	conserved fungal protein	protein_coding	II	1
41	SPBC651.01c	GTP binding protein Nog1 (predicted)	protein_coding	II	-1
42	SPBC428.10	sequence orphan	protein_coding	II	1
42	SPCC1450.07c	D-amino acid oxidase	protein_coding	III	-1
43	SPCC162.04c	wtf element Wtf13	protein_coding	III	-1
43	SPCC1906.03	wtf element Wtf19	protein_coding	III	1
43	SPCC548.03c	wtf element Wtf4	protein_coding	III	-1
44	SPCC330.10	P-TEFb-cap methyltransferase Pcm1	protein_coding	III	1
44	SPCC645.13	transcription elongation regulator (predicted)	protein_coding	III	1
45	SPNCRNA.210	non-coding RNA (predicted)	ncRNA	I	1
45	SPNCRNA.865	antisense RNA (predicted)	ncRNA	I	1
46	SPNCRNA.491	non-coding RNA (predicted)	ncRNA	III	1
46	SPSNORNA.54	SnoRNA (predicted)	snoRNA	I	1
47	SPNCRNA.727	intergenic RNA (predicted)	ncRNA	I	1
47	SPSNORNA.10	small nucleolar RNA Z16 (predicted)	snoRNA	I	1

Table S4: mRNAs that behaved strongly proportionally

References

[1] Friedrich Leisch. "Sweave: Dynamic Generation of Statistical Reports Using Literate Data Analysis". In: *Compstat 2002 — Proceedings in Computational Statistics*. Ed. by Wolfgang Härdle and Bernd Rönz. ISBN 3-7908-1517-9. Physica Verlag, Heidelberg, 2002, pp. 575–580.

[2] Yihui Xie. *knitr: A general-purpose package for dynamic report generation in R*. R package version 1.5. 2013.

[3] Samuel Marguerat et al. "Quantitative Analysis of Fission Yeast Transcriptomes and Proteomes in Proliferating and Quiescent Cells". In: *Cell* 151.3 (Oct. 2012), pp. 671–683. ISSN: 0092-8674. DOI: `10.1016/j.cell.2012.09.019`. (Visited on 12/01/2012).

[4] K. Gerald van den Boogaart, Raimon Tolosana, and Matevz Bren. *compositions: Compositional Data Analysis*. R package version 1.30-2. 2013.

[5] S original by David W. Scott R port by Albrecht Gebhardt adopted to recent S-PLUS by Stephen Kaluzny ¡spk@insightful.com¿. *ash: David Scott's ASH routines*. R package version 1.0-14. 2013.

[6] R Core Team. *R: A Language and Environment for Statistical Computing*. R Foundation for Statistical Computing. Vienna, Austria, 2013.

[7] Ross Ihaka et al. *colorspace: Color Space Manipulation*. R package version 1.2-4. 2013.

[8] Hadley Wickham and Winston Chang. *ggplot2: An implementation of the Grammar of Graphics*. R package version 0.9.3.1. 2013.

[9] Gregory R. Warnes et al. *gplots: Various R programming tools for plotting data*. R package version 2.12.1. 2013.

[10] Baptiste Auguie. *gridExtra: functions in Grid graphics*. R package version 0.9.1. 2012.

[11] Gabor Csardi. *igraph: Network analysis and visualization*. R package version 0.7.0. 2014.

[12] Brian Ripley. *MASS: Support Functions and Datasets for Venables and Ripley's MASS*. R package version 7.3-31. 2014.

[13] Hadley Wickham. *plyr: Tools for splitting, applying and combining data*. R package version 1.8.1. 2014.

[14] Henrik Bengtsson. *R.methodsS3: Utility function for defining S3 methods*. R package version 1.6.1. 2014.

[15] Henrik Bengtsson. *R.oo: R object-oriented programming with or without references*. R package version 1.18.0. 2014.

[16] Henrik Bengtsson. *R.utils: Various programming utilities*. R package version 1.29.8. 2014.

[17] Erich Neuwirth. *RColorBrewer: ColorBrewer palettes*. R package version 1.0-5. 2011.

[18] Hadley Wickham. *reshape: Flexibly reshape data*. R package version 0.8.4. 2011.

[19] Hadley Wickham. *scales: Scale functions for graphics*. R package version 0.2.3. 2012.

[20] David Warton et al. *smatr: (Standardised) Major Axis Estimation and Testing Routines*. R package version 3.4-3. 2014.

[21] K. Gerald van den Boogaart. *tensorA: Advanced tensors arithmetic with named indices*. R package version 0.36. 2010.

[22] David B. Dahl. *xtable: Export tables to LaTeX or HTML*. R package version 1.7-3. 2014.

[23] John Aitchison. "A Concise Guide to Compositional Data Analysis". In: *2nd Compositional Data Analysis Workshop*. Girona, Italy, 2003.

[24] Jakob Lovén et al. "Revisiting Global Gene Expression Analysis". In: *Cell* 151.3 (Oct. 2012), pp. 476–482. ISSN: 0092-8674. DOI: `10.1016/j.cell.2012.10.012`. (Visited on 10/31/2012).

[25] Subhadeep Mukhopadhyay and Emanuel Parzen. *LP Approach to Statistical Modeling*. 2014. URL: `http://arxiv.org/pdf/1405.2601.pdf`.

[26] Xiao Xiao et al. “On the use of log-transformation vs. nonlinear regression for analyzing biological power laws”. In: *Ecology* 92.10 (June 2011), pp. 1887–1894. ISSN: 0012-9658. DOI: 10.1890/11-0538.1. (Visited on 02/01/2014).

[27] Dominic O’Neil, Heike Glowatz, and Martin Schlumpberger. “Ribosomal RNA Depletion for Efficient Use of RNA-Seq Capacity”. en. In: *Current Protocols in Molecular Biology*. John Wiley & Sons, Inc., 2001. ISBN: 9780471142720. (Visited on 12/30/2013).

[28] J. Aitchison. *The statistical analysis of compositional data*. Chapman & Hall, Ltd., 1986.

[29] David I. Warton et al. “Bivariate line-fitting methods for allometry”. en. In: *Biological Reviews* 81.2 (2006), pp. 259–291. ISSN: 1469-185X. DOI: 10.1017/S1464793106007007. (Visited on 01/19/2013).

[30] Jonathan Friedman and Eric J. Alm. “Inferring Correlation Networks from Genomic Survey Data”. In: *PLoS Comput Biol* 8.9 (Sept. 2012), e1002687. DOI: 10.1371/journal.pcbi.1002687. (Visited on 10/24/2012).

[31] S. S. Stevens. “On the Theory of Scales of Measurement”. en. In: *Science* 103.2684 (June 1946), pp. 677–680. ISSN: 0036-8075, 1095-9203. DOI: 10.1126/science.103.2684.677. (Visited on 02/02/2014).

[32] Vera Pawlowsky-Glahn, Juan José Egozcue, and David Roger Lovell. “Tools for compositional data with a total”. In: *Statistical Modelling* (2014). To appear.
